# Esterase-Responsive Mitochondria-Targeted Hydropersulfide Donors Mitigate Doxorubicin Cardiotoxicity While Preserving Anticancer Activity

**DOI:** 10.1002/anie.202521645

**Published:** 2025-12-12

**Authors:** Jinjing Gu, Qi Liu, Deborah Rodriguez, Jordan Lamar, Klaire R. Bradley, Gizem Keceli, Andrew Thampoe, Yihang Xiao, Nazareno Paolocci, Vinayak S. Khodade, John P. Toscano

**Affiliations:** Department of Chemistry, Johns Hopkins University, Baltimore, Maryland 21210, USA; Department of Chemistry, Johns Hopkins University, Baltimore, Maryland 21210, USA; Department of Chemistry, Johns Hopkins University, Baltimore, Maryland 21210, USA; Department of Chemistry, Johns Hopkins University, Baltimore, Maryland 21210, USA; Department of Chemistry, Johns Hopkins University, Baltimore, Maryland 21210, USA; Division of Cardiology, Johns Hopkins University School of Medicine, Baltimore, Maryland 21205, USA; Department of Chemistry, Johns Hopkins University, Baltimore, Maryland 21210, USA; Department of Chemistry, Johns Hopkins University, Baltimore, Maryland 21210, USA; Division of Cardiology, Johns Hopkins University School of Medicine, Baltimore, Maryland 21205, USA; Department of Biomedical Sciences, University of Padova, Padova, 35131, Italy; Department of Chemistry, Johns Hopkins University, Baltimore, Maryland 21210, USA; Smidt Heart Institute, Department of Cardiac Surgery, Cedars-Sinai, Medical Center, Los Angeles, CA 90048, USA; Department of Chemistry, Johns Hopkins University, Baltimore, Maryland 21210, USA

**Keywords:** Cardio-oncology, Doxorubicin cardiotoxicity, Mitochondria function, Mitochondria-targeted hydropersulfide donors, Myocardial redox balance

## Abstract

Therapeutic agents that protect the heart from doxorubicin (DOX) toxicity without reducing its anticancer efficacy remain a critical unmet need. We report esterase-activated hydropersulfide (RSSH) donors, alkyl sulfenyl thiocarbonate (**AST-2**), and acetoxy perthiocarbamate (**APT-1**), together with their mitochondria-targeted analogs, **AST-2-TPP** and **APT-1-TPP**, which bear a triphenylphosphonium (TPP^+^) moiety. These compounds release RSSH upon esterase activation with tunable half-lives (20–125 min in PBS, pH 7.4). LC–MS/MS analysis revealed that **APT-1** elevates hydropersulfide levels in the cytosol of H9c2 cardiomyoblasts, whereas its mitochondrial analog, **APT-1-TPP**, increases levels in mitochondria. All donors attenuated DOX-induced toxicity in H9c2 cells, but in cancer cell lines (HepG2, MDA-MB-468, MCF-7), **APT-1** did not blunt DOX cytotoxicity and **APT-1-TPP** synergistically enhanced its activity. Mechanistic studies revealed that both **APT-1** and **APT-1-TPP** rescue DOX-induced mitochondrial membrane depolarization and ATP depletion in H9c2 cells but not in HepG2 cells. Further characterization indicated that cancer cells exhibit higher basal sulfane sulfur levels and mitochondrial membrane potentials compared to H9c2 cells, suggesting that divergent redox environments may underlie these contrasting effects. Collectively, these findings demonstrate that redox heterogeneity between cardiac and cancer cells can be exploited to develop cardioprotective interventions that preserve or enhance DOX’s anticancer efficacy.

## Introduction

Doxorubicin (DOX) remains a cornerstone chemotherapeutic for a broad spectrum of malignancies. However, its clinical utility is limited by cumulative, dose-dependent cardiotoxicity, which often results in irreversible cardiac dysfunction and progression to heart failure.^[1,2]^ This presents a serious and unresolved challenge in oncology. Although cardioprotective strategies such as iron chelation (e.g., dexrazoxane) and antioxidant therapies have been explored, their impact is constrained by limited efficacy and undesirable attenuation of DOX’s anticancer activity.^[3,4]^ Thus, there is an urgent need for cardioprotective interventions that preserve myocardial function without compromising the therapeutic efficacy of DOX.

DOX-induced cardiotoxicity is primarily driven by the excessive generation of reactive oxygen species (ROS) through multiple mechanisms.^[5]^ Mitochondria have emerged as central mediators of this toxicity, acting both as major ROS sources and as primary damage targets.^[6]^ Elevated ROS impairs mitochondrial oxidative phosphorylation, disrupts membrane potential, promotes opening of the mitochondrial permeability transition pore, and triggers energetic collapse.^[7–11]^ Mitochondrial dysfunction, in turn, activates intrinsic apoptotic pathways, ultimately leading to cardiomyocyte death.^[12]^

**Scheme 1.**
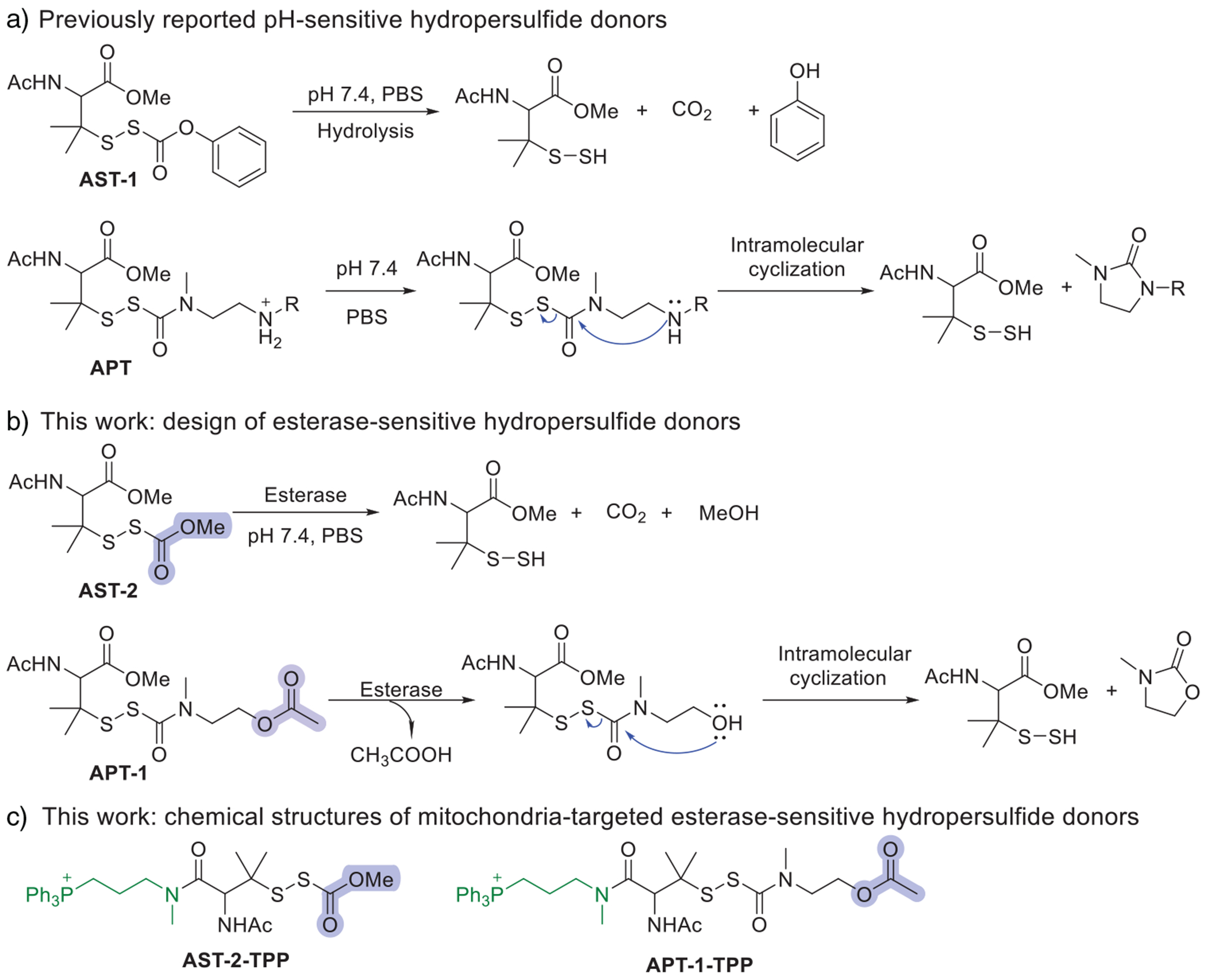
Design of esterase-sensitive RSSH donors.

Reactive sulfur species have emerged as promising candidates to counteract these effects due to their ability to modulate redox-sensitive signaling pathways.^[13]^ For example, multiple studies have demonstrated that hydrogen sulfide (H_2_S) protects cardiomyocytes from DOX-induced mitochondrial injury and ferroptosis.^[14–16]^ Notably, H_2_S-donating DOX hybrids have been reported to attenuate DOX-induced cardiotoxicity.^[17,18]^ More recently, hydropersulfides (RSSH) have attracted attention as potent cardioprotective agents.^[19,20]^ Compared to H_2_S, RSSH possess greater nucleophilic and antioxidant capacity, enabling them to scavenge ROS more efficiently.^[21]^ In addition, RSSH can activate endogenous antioxidant pathways. We have shown that the RSSH donor alkylsulfenyl perthiocarbonate (**AST-1**) protects cardiomyocytes against DOX-induced oxidative injury.^[22,23]^ This protection is mediated, in part, through activation of the nuclear factor erythroid 2–related factor 2 (Nrf2) endogenous antioxidant pathway and promotion of mitochondrial biogenesis via peroxisome proliferator–activated receptor gamma coactivator 1-alpha (PGC-1*α*) upregulation. In addition, we reported a class of pH-sensitive alkylamine-substituted perthiocarbamate (**APT**) donors that release RSSH upon neutralization of the terminal ammonium group under physiological conditions, followed by intramolecular cyclization (Scheme 1a).^[24]^ These donors demonstrated cardioprotective effects in both in vitro (H9c2 cardiomyocytes) and ex vivo (murine ischemia-reperfusion) models.^[25]^ Despite these promising results, both **AST-1** and **APT** are limited by their reliance on pH-dependent activation, which restricts control of RSSH release. Furthermore, **AST-1** suffers from hydrolytic instability and poor aqueous solubility.

To overcome these challenges, we sought to develop esterase-responsive RSSH donors with temporal control over delivery and improved aqueous stability. Esterases are attractive biological triggers due to their broad substrate specificity and elevated activity in metabolically active organs such as the heart.^[26,27]^ Importantly, esterase-mediated activation occurs intracellularly,^[28]^ enabling localized RSSH release within cells. Several esterase-sensitive RSSH donors have been reported, exhibiting protective effects against oxidative stress and myocardial ischemia–reperfusion injury.^[29–33]^ However, these systems lack organelle-specific targeting. To this end, we designed two esterase-responsive donors. The first, **AST-2**, is a structural analog of **AST-1** in which the phenyl ring is replaced by a methyl group (Scheme 1b). This modification reduces the electrophilicity of the carbonyl group, thereby improving hydrolytic stability while maintaining susceptibility to esterase-mediated activation. Second, we modified the **APT** scaffold by introducing an esterase-cleavable acetyl group in place of the terminal amine (Scheme 1b). Upon esterase-mediated cleavage, the resulting hydroxyl intermediate is expected to undergo spontaneous intramolecular cyclization, leading to RSSH release.

Given the central role of mitochondrial oxidative stress in DOX-induced cardiotoxicity,^[34]^ we also sought to target these donors specifically to the mitochondria. The triphenylphosphonium (TPP^+^) moiety is a well-established lipophilic cation that targets mitochondria due to their negative transmembrane potential.^[35]^ Conjugation of small-molecule therapeutics to TPP^+^ has been widely employed to achieve mitochondrial targeting.^[35]^ For example, Whiteman and co-workers demonstrated that the mitochondria-targeted hydrogen sulfide (H_2_S) donor AP39 more potently inhibits oxidative stress-induced cell death in hCMEC/D3 cells than the non-targeted H2S donor GYY4137.^[36]^ Recently, Murphy and co-workers developed MitoPerSulf, an H_2_S donor that rapidly and selectively generates H_2_S within mitochondria, and demonstrated that it protects against myocardial ischemia–reperfusion injury.^[37]^ Moreover, Chakrapani and co-workers reported a novel artificial substrate for mitochondrial 3-mercaptopyruvate sulfurtransferase (3-MST) and demonstrated efficient generation of sulfane sulfur that modulated mitochondrial redox state and membrane potential.^[38]^ Based on these findings, we hypothesized that mitochondrial targeting could similarly enhance the efficacy of RSSH donors. We conjugated TPP^+^ to each RSSH scaffold, producing **AST-2-TPP** and **APT-1-TPP** (Scheme 1c). In this design, the TPP^+^ moiety is linked to the RSSH core via a stable amide linkage, rather than to the protecting group, ensuring that RSSH mitochondrial targeting is retained following enzymatic activation.

## Results and Discussion

### Synthesis of Esterase-Sensitive Hydropersulfide Precursors and Hydropersulfide Release Studies

We synthesized **AST-2** as an esterase-sensitive RSSH donor via reaction of *N*-acetylpenicillamine methyl ester with methoxycarbonyl sulfenyl chloride in dichloromethane at 0 °C, affording the product in 72% yield (see Scheme S1). We first evaluated the aqueous stability of **AST-2** in phosphate-buffered saline (PBS, pH 7.4) at 37 °C. Compared to the previously reported analogue **AST-1** (complete degradation over 9 h), **AST-2** demonstrates enhanced stability, with only 22% decomposition under identical conditions (SI, Figure S1A).

**Figure 1.**
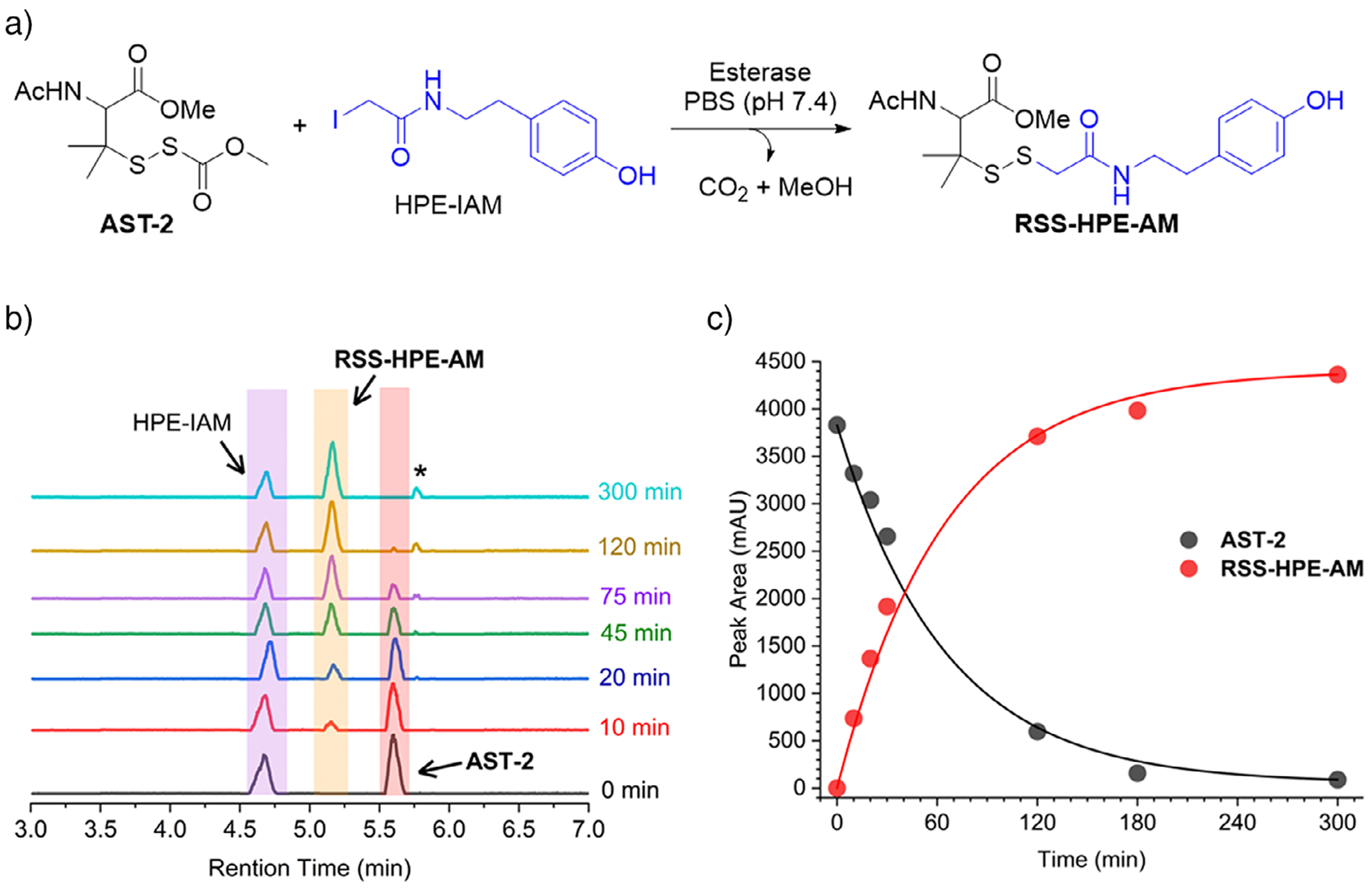
a) Schematic of esterase-mediated RSSH release from **AST-2** in the presence of the trapping agent HPE-IAM. b) Representative UPLC-MS traces showing time-dependent conversion of **AST-2** (25 μM) to **RSS-HPE-AM** upon treatment with porcine liver esterase (1 U mL^−1^) and HPE-IAM (250 μM) in 100 mM ammonium bicarbonate buffer (pH 7.4) containing the metal chelator diethylenetriaminepentaacetic acid (DTPA) (100 μM) at 37 °C. Aliquots were quenched with formic acid and analyzed by UPLC-MS. Asterisk indicates a minor dialkyltrisulfide byproduct. c) First-order kinetic analysis of **AST-2** consumption (*k* = 0.0139 ± 0.0005 min^−1^, *t*_1/2_ = 50.1 ± 1.8 min) and **RSS-HPE-AM** formation (*k* = 0.0137 ± 0.0006 min^−1^, *t*_1/2_ = 48.3 ± 2.1 min). Rate constants and half-lives are reported as mean ± SD (*n* = 3). Curves represent best fits to single-exponential functions.

We then assessed esterase-mediated activation of **AST-2** by incubating it with porcine liver esterase (PLE) in the presence of *β*-(4-hydroxyphenyl)ethyl iodoacetamide (HPE-IAM, 10 equiv), a trapping agent for RSSH.^[39]^ We anticipated that PLE-catalyzed hydrolysis would release RSSH, which would subsequently be trapped by HPE-IAM to generate **RSS-HPE-AM** (Figure 1a). As expected, UPLC-MS analysis reveals complete consumption of **AST-2**, accompanied by the appearance of **RSS-HPE-AM**, confirming esterase-triggered RSSH release (Figures 1b and S3). In addition, a minor amount of dialkyl trisulfide formation is observed (Figures 1b and S5), which we attribute to the reaction of the released RSSH with the precursor **AST-2**. Kinetic analysis reveals first-order behavior, with a rate constant of *k* = 0.0139 ± 0.0005 min^−1^ and a half-life (*t*_1/2_) = 50.1 ± 1.8 min for **AST-2** decay. The concomitant formation of **RSS-HPE-AM** proceeded with a rate constant (*k* = 0.0144 ± 0.0006 min^−1^, *t*_1/2_ = 48.3 ± 2.1 min), indicating that consumption of **AST-2** is the rate-determining step and trapping of the released RSSH is fast under our experimental conditions (Figure 1c). Quantitative analysis revealed 95% formation of **RSS-HPE-AM**.

Encouraged by these results, we next investigated incorporating an esterase-responsive trigger onto the **APT** scaffold to extend the half-life of the precursor and potentially enhance bioactivity. **APT-1** was synthesized in 93% overall yield via a one-pot two-step sequence: *N*-acetylpenicillamine methyl ester was reacted with chlorocarbonylsulfenyl chloride to afford an S-perthiocarbonyl chloride intermediate, which was subsequently treated with 2-acetoxy-*N*-methylethan-1-aminium chloride in the presence of triethylamine (see Supporting Information). **APT-1** exhibits excellent stability in PBS at 37 °C, with only 7% decomposition over 48 h (Figure S1B).

**Figure 2.**
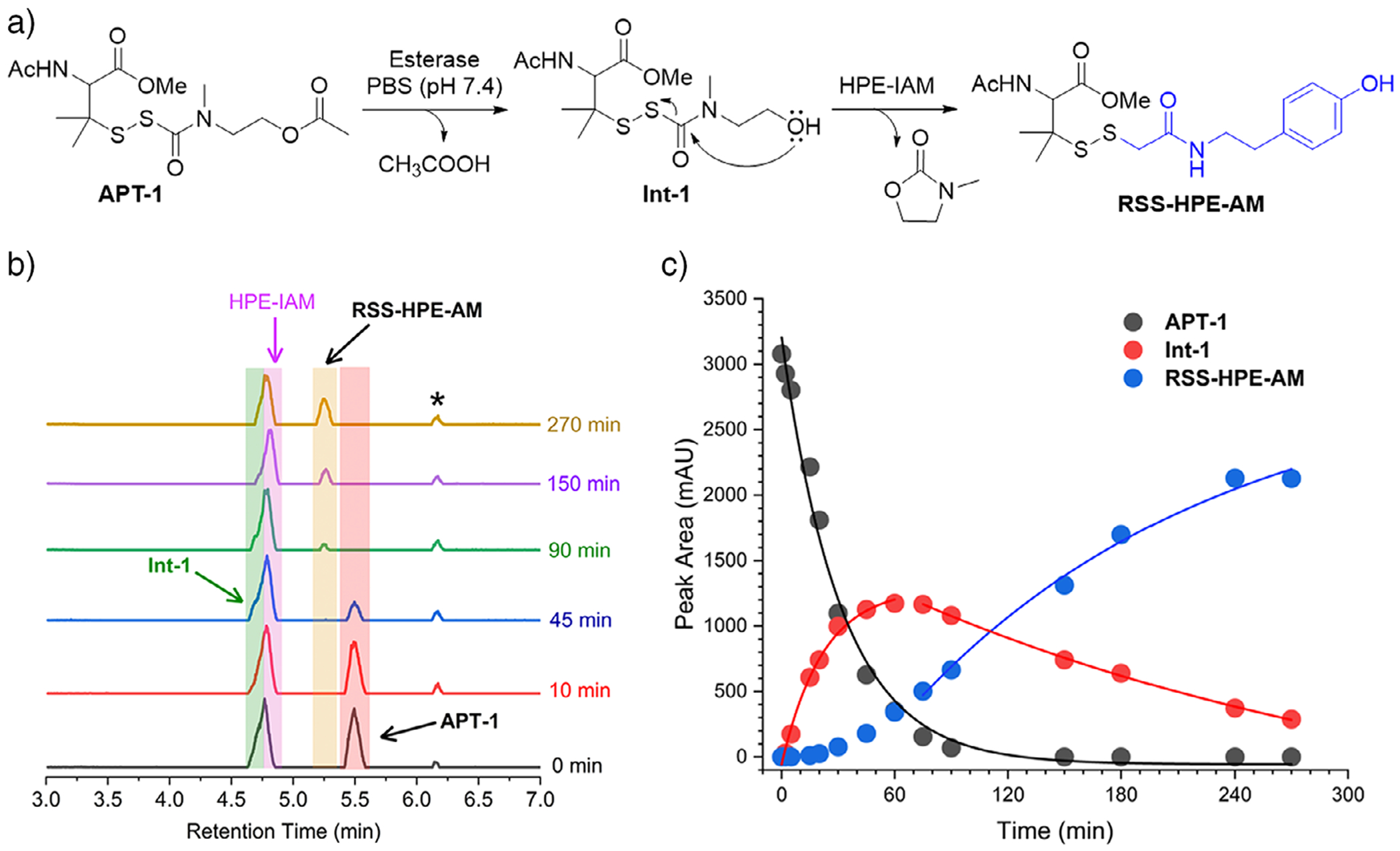
a) Reaction scheme showing esterase-triggered RSSH release from **APT-1** via a two-step activation involving deacetylation and intramolecular cyclization. b) Representative UPLC-MS traces showing the time-dependent conversion of **APT-1** (25 μM) to intermediate **1** and subsequent formation of **RSS-HPE-AM** in the presence of PLE (1 U mL^−1^) and HPE-IAM (250 μM) at 37 °C. Asterisk indicates a minor impurity in the blank control. Although the **Int-1** peak partially overlaps with the HPE-IAM signal in the base peak intensity (BPI) chromatogram, extracted-ion chromatograms (EICs) based on exact mass enable selective detection of **Int-1**, as shown in Figure S6. c) Kinetic analysis of rapid consumption of **APT-1** (*k*_1_ = 0.0406 ± 0.0010 min^−1^, *t*_1/2_ = 17.1 ± 0.4 min), formation and consumption of **Int-1** and **RSS-HPE-AM** formation (*k*_2_ = 0.0067 ± 0.0006 min^−1^, *t*_1/2_ = 104.9 ± 9.8 min). Rate constants and half-lives are reported as mean ± SD (*n* = 3). Curves represent best fits to single exponential functions as described in the text.

Upon incubation with PLE and HPE-IAM, UPLC–MS analysis revealed rapid disappearance of the **APT-1** peak with concomitant formation of a new peak corresponding to the alcohol intermediate (**Int-1**), which gradually converted to **RSS-HPE-AM** (Figure 2a,b). Notably, the kinetics of **RSS-HPE-AM** formation is consistent with rapid esterase-mediated deacetylation to form **Int-1**, followed by slower intramolecular cyclization to release RSSH (Figure 2c). Kinetic analysis showed that deacetylation proceeded with a first-order rate constant of *k*_1_ = 0.0406 ± 0.0010 min^−1^ (*t*_1/2_ = 17.1 ± 0.4 min), whereas cyclization-mediated RSSH release was slower (*k*_2_ = 0.0067 ± 0.0006 min^−1^, *t*_1/2_ = 104.9 ± 9.8 min). HPLC analysis showed 92% formation of **RSS-HPE-AM**, confirming the high efficiency of this precursor.

### Synthesis of Esterase-Sensitive Mitochondria Targeted Hydropersulfide Precursors and Release Studies

**Scheme 2.**
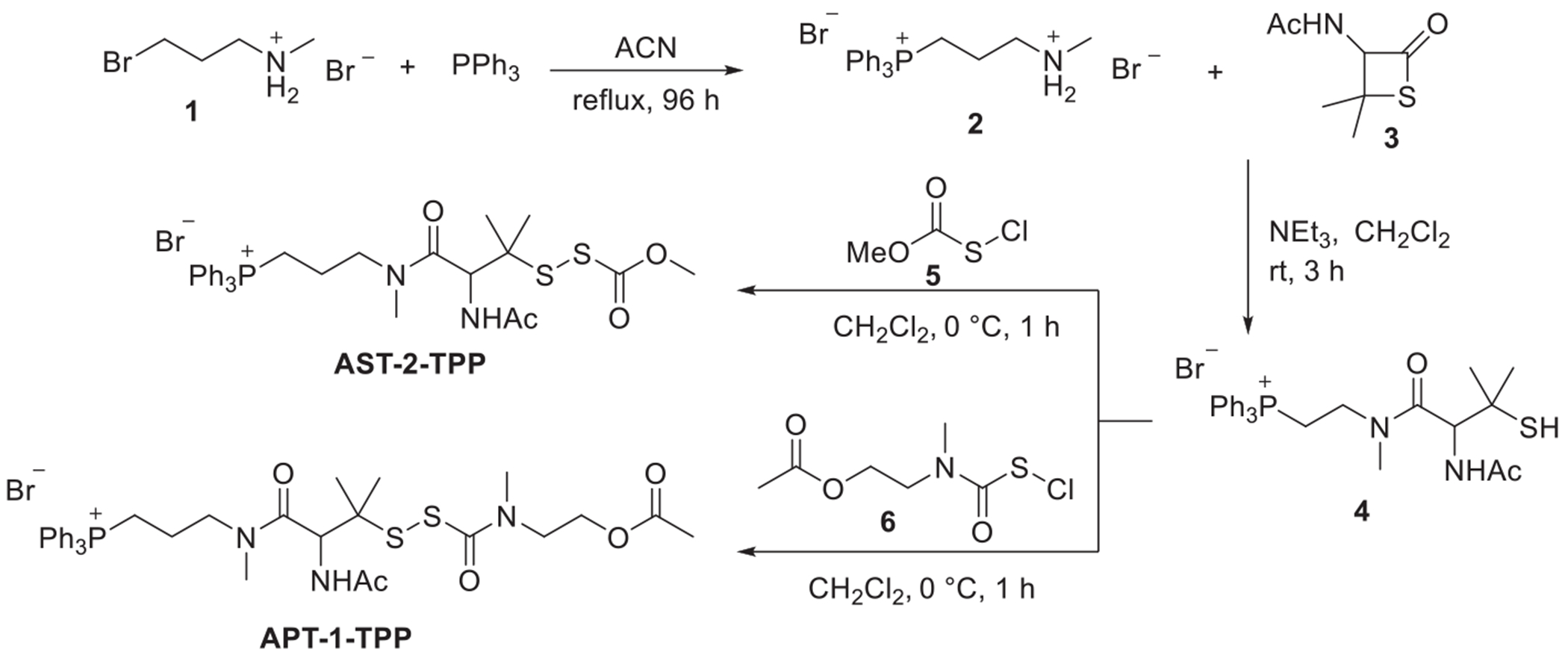
Synthesis of mitochondria-targeted esterase-responsive RSSH donors **AST-2-TPP** and **APT-1-TPP**.

We next incorporated the TPP^+^ moiety into the **AST-2** and **APT-1** scaffolds to enable mitochondrial targeting. To ensure hydrolytic stability, the TPP^+^ unit was introduced via an amide linkage, with a three-carbon aliphatic spacer between TPP^+^ and the penicillamine core to minimize steric hindrance. As outlined in Scheme 2, the synthesis of **AST-2-TPP** commenced with the nucleophilic substitution of 3-bromo-*N*-methylpropanamine hydrobromide (**1**) with triphenylphosphine in refluxing acetonitrile, which furnished the key intermediate [3-(methylamino)propyl]triphenylphosphonium bromide (**2**) with an 81% yield. Subsequent reaction of**2** with thiolactone **3** produced the TPP-functionalized penicillamine **4** (64%), which was then treated with methoxycarbonyl sulfenyl chloride (**5**) at 0 °C to yield **AST-2-TPP** (43%). Similarly, **APT-1-TPP** was prepared by reacting **4** with sulfenyl chloride intermediate **6** in dichloromethane, affording **APT-1-TPP** in 30% yield. (See Supporting Information for full experimental procedures and characterization data.)

We first evaluated the aqueous stability of the mitochondria-targeted analogs **AST-2-TPP** and **APT-1-TPP**. In PBS (pH 7.4, 37 °C), **AST-2-TPP** exhibits comparable stability to its non-targeted analogue, showing ca. 20% decomposition over 7 h versus 22% for **AST-2** over 9 h (Figure S1C). **APT-1-TPP** similarly retained excellent stability, with < 5% degradation over 41 h (Figure S1D). This data shows that TPP conjugation does not compromise the aqueous stability of these precursors.

UPLC-MS analysis confirms efficient RSSH release from **AST-2-TPP** in the presence of PLE, evidenced by the disappearance of the precursor peak and concomitant formation of **TPP-RSS-HPE-AM** (Figure S11). Interestingly, the release kinetics are significantly accelerated compared to **AST-2**, with a first-order rate constant of *k* = 0.0347 ± 0.0028 min^−1^ (*t*_1/2_ = 20.1 ± 1.6 min). This rate enhancement suggests that the TPP moiety, despite its spatial separation from the ester group, may influence substrate–enzyme interactions. For quantification, we synthesized an authentic standard of **TPP-RSS-HPE-AM** (see Scheme S3). Quantitative analysis revealed 91% formation of **TPP-RSS-HPE-AM** (Table 1).

**APT-1-TPP** follows a two-step activation mechanism analogous to **APT-1**. Upon PLE treatment, rapid deacetylation occurs with *k*_1_ = 0.134 ± 0.005 min^−1^ (*t*_1/2_ = 5.2 ± 0.2 min) (Figure S15), which is significantly faster than that of its non-TPP analogue. The positive charge on TPP moiety may contribute to stabilization of the tetrahedral intermediate formed in the transition state, leading to a lowered activation energy and faster observed rate. While the exact molecular basis for this rate enhancement remains to be determined, it may arise from physicochemical factors such as improved solubility, enhanced substrate binding affinity, or altered substrate orientation within the enzyme’s active site, all of which can influence catalytic efficiency. The subsequent cyclization-mediated RSSH release step proceeds relatively slowly (*k*_2_ = 0.0056 ± 0.0004 min^−1^, *t*_1/2_ = 124.8 ± 9.4 min), compared with **APT-1**, yet overall conversion remains high (95%). These results indicate that TPP conjugation preserves efficient enzymatic activation while modulating the kinetics of hydrolysis and cyclization, ultimately maintaining high RSSH release efficiency without compromising donor stability.

### Stability of Hydropersulfide Precursors Toward Thiols

**Scheme 3.**
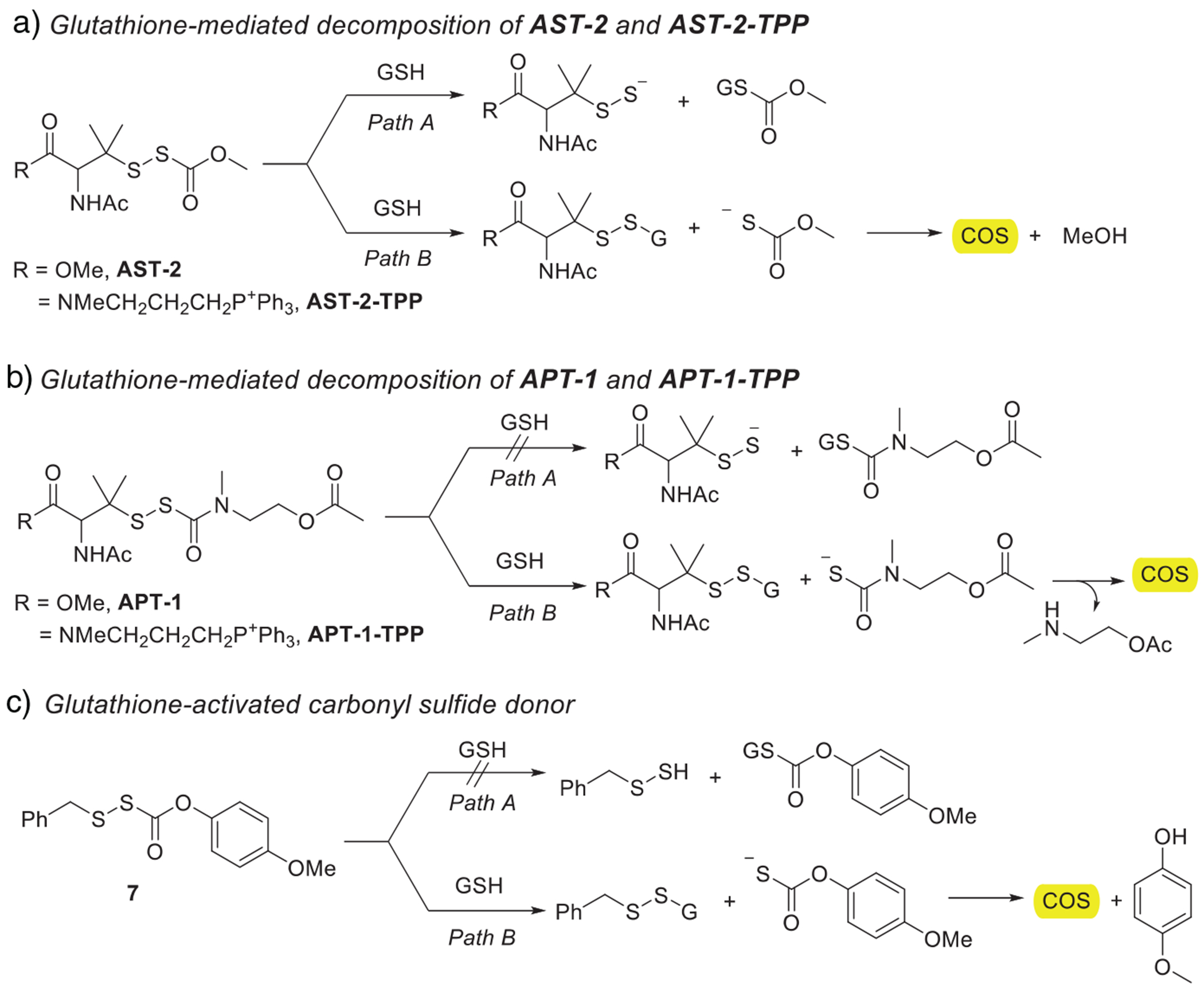
Proposed thiol-mediated decomposition pathways of **AST** and **APT** RSSH precursors.

We next evaluated whether our newly developed precursors generate RSSH in the presence of thiols, which are abundant in biological systems. We examined their reactivity toward glutathione (GSH, 5 equiv) in PBS (pH 7.4, 37 °C), and precursor consumption was quantified by HPLC. All four precursors underwent thiol-dependent degradation, though at notably different rates (Figure S19). **AST-2** and **AST-2-TPP** were consumed quickly (*t*_1/2_ = 9 and 6 min, respectively), whereas **APT-1** and **APT-1-TPP** reacted much more slowly (*t*_1/2_ = 40 and 56 min, respectively). We attribute this difference to their intrinsic reactivity. As shown previously,^[23]^ **AST** donors can undergo nucleophilic attack either at the carbonyl carbon, producing the intended RSSH and a thiocarbonate byproduct (Scheme 3a, Path A), or at the disulfide bond, giving an unsymmetrical disulfide and a thiocarbonate intermediate (Scheme 3a, Path B). The higher electrophilicity of the perthiocarbonate carbonyl likely explains the faster consumption of **AST** derivatives. In contrast, and consistent with earlier studies,^[24]^ **APT-1** and **APT-1-TPP** react mainly through thiol–disulfide exchange (Scheme 3b, Path B), as attack at the perthiocarbamate carbonyl is disfavored due to its lower electrophilicity compared with the perthiocarbonate in **AST** donors.

To probe the GSH-mediated decomposition pathway, we monitored carbonyl sulfide (COS) formation, which reports on the disulfide-exchange route. Thiol-mediated disulfide exchange generates a thiocarbonate (from **AST**) or thiocarbamate (from **APT**) intermediate, and both can decompose to release COS (Scheme 3, Path B). COS generation was measured in real time by membrane inlet mass spectrometry (MIMS).^[23]^ As a reference, we used compound **7**, which is known to react exclusively through disulfide exchange and to release COS efficiently (Scheme 3c). Compound **7** showed rapid COS release under these conditions (Figure S20). The RSSH precursors also produced detectable COS upon GSH treatment, but both the rates and overall yields were substantially lower (ca.10–20%) than those of compound **7** (Figure S20). This reduction suggests that disulfide exchange occurs but is not the dominant pathway for these donors. The steric bulk of the gem-dimethyl group adjacent to the disulfide likely slows thiol attack and contributes to the reduced COS yield.

Overall, the precursors are reactive toward thiols, but the two donor classes behave differently. **AST** derivatives are consumed more quickly, yet their modest COS yields imply that RSSH formation is still the major decomposition route. **APT** donors react more slowly with GSH and therefore remain available for esterase-mediated activation to release RSSH. Finally, any COS formed intracellularly would be rapidly hydrolyzed by carbonic anhydrase to produce H_2_S, another relevant reactive sulfur species.

### APT-1 Elevates Cytosolic Hydropersulfides, While APT-1-TPP Elevates Mitochondrial Hydropersulfides

**Figure 3.**
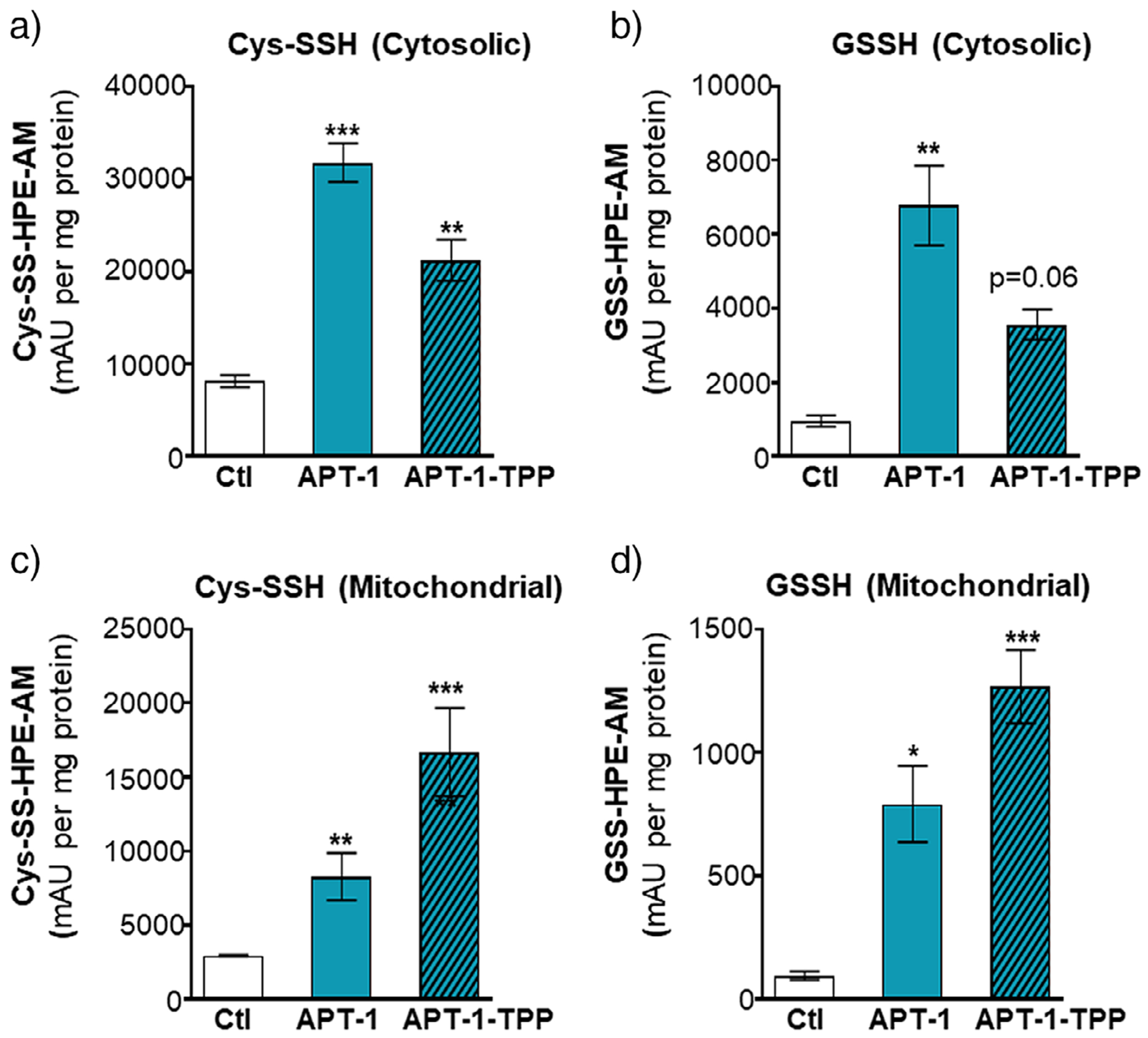
Measurement of cysteine hydropersulfide (Cys-SSH) and glutathione hydropersulfide (GSSH) in cytoplasm (a and b) and mitochondria (c and d) of H9c2 cells. Cells were treated with **APT-1** (200 μM), **APT-1-TPP** (200 μM), or vehicle (0.01% DMSO in serum-free medium) for 2 h, followed by subcellular fractionation. Each fraction was incubated with HPE-IAM (5 mM, 30 min) to trap RSSH species prior to LC–MS/MS analysis. Data are mean ± SEM (*n* = 3); *p ≤ 0.05, **p ≤ 0.01.

After confirming efficient esterase-triggered RSSH generation from these newly developed precursors in PBS (pH 7.4), we next evaluated their ability to release RSSH in cardiac cells. For this study, we chose the **APT-1**/**APT-1-TPP** donor pair due to their extended half-lives and superior aqueous stability. H9c2 cells were treated with each donor (200 μM, 2 h), followed by subcellular fractionation using differential centrifugation to isolate cytoplasmic and mitochondrial fractions. Each fraction was subsequently incubated with the RSSH-trapping agent HPE-IAM (5 mM in methanol) to alkylate and stabilize transient RSSH species. The resulting adducts were analyzed by LC–MS/MS.^[40,41]^ In the cytoplasmic fraction, both donors increased HPE-AM adducts of Cys-SSH and GSSH, with **APT-1** showing higher levels than **APT-1-TPP** (Figure 3a,b). In contrast, the mitochondria-targeted donor **APT-1-TPP** produced substantially higher levels of Cys-SSH and GSSH adducts in the mitochondrial fraction compared with **APT-1** (Figure 3c,d). Interestingly, no precursor-derived **RSS-HPE-AM** or **TPP-RSS-HPE-AM** were detectable, indicating that the sterically hindered tertiary RSSH species initially generated rapidly transpersulfidates endogenous thiols such as cysteine and glutathione.^[42]^

In addition to Cys-SSH and GSSH, we also observed elevated H_2_S and H_2_S_2_ levels in the cytoplasm with **APT-1** treatment and in mitochondria with **APT-1-TPP** treatment (Figure S21). The presence of these species aligns with previous reports showing that RSSH are chemically unstable under cellular conditions, undergoing disproportionation and interconversion to other reactive sulfur species.^[39]^ Under our experimental conditions, HPE-IAM is added after donor incubation, during which disproportionation likely occurs prior to trapping. Of note, a portion of the H_2_S may also arise from the COS intermediate formed via thiol-mediated precursor decomposition. In addition, we observed significant increases in Cys-SH and GSH in the cytoplasm upon treatment with **APT-1** and **APT-1-TPP**, whereas in mitochondria, modest increases were observed with both donors that did not reach statistical significance (Figure S22)

Given the intracellular abundance of thiols, donor consumption through thiol–disulfide exchange or premature ester hydrolysis in the cytosol could potentially interfere with mitochondrial targeting. However, the relatively slow thiol reactivity of **APT** precursors observed in buffer appears to extend to the cellular environment, allowing the donors to remain intact long enough for esterase activation. Even if some activation occurs prior to mitochondrial entry, the resulting TPP-linked RSSH intermediate released is expected to accumulate in mitochondria due to the strong membranepotential-driven uptake of TPP scaffolds. Indeed, lipophilic TPP conjugates are reported to concentrate in mitochondria within seconds to minutes driven by the mitochondrial membrane potential,^[43,44]^ suggesting that mitochondrial uptake is likely favored over competing thiol-mediated consumption in the cytosol. Collectively, these results demonstrate that **APT-1** and **APT-1-TPP** are cell-permeable and activated in H9c2 cells, and that conjugation with triphenylphosphonium substantially enhances mitochondrial targeting of RSSH delivery.

### Hydropersulfide Donors Protect H9c2 Cells Against Doxorubicin-Induced Toxicity

**Figure 4.**
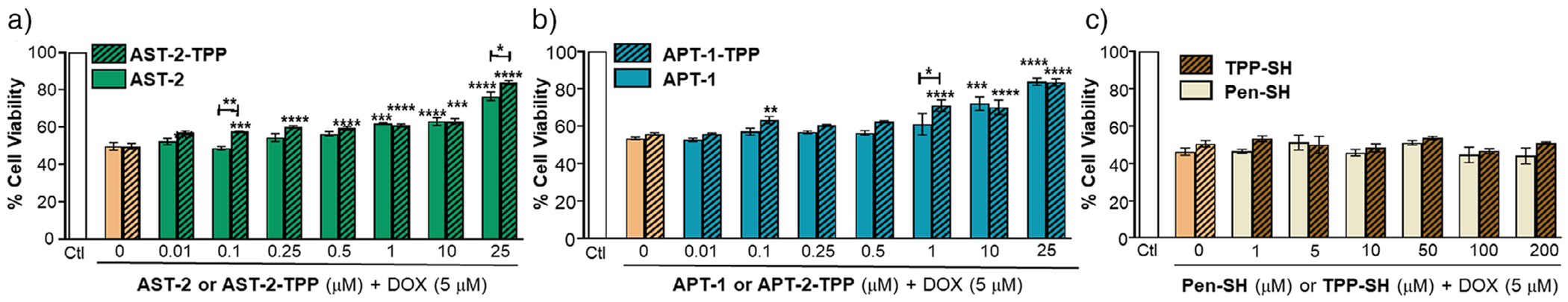
Protective effects of RSSH donors against DOX-induced cytotoxicity in H9c2 cells. Cells were pretreated with RSSH donors for 4 h, followed by exposure to DOX (5 μM) for an additional 24 h. Cell viability was assessed using the cell counting Kit-8 (CCK-8) assay. Results are expressed as mean ± SEM, *n* = 3; *P < 0.05, **P < 0.01, ***P < 0.001, ****P < 0.0001. Ctl = vehicle-treated group (0.01% DMSO in medium).

We evaluated the cytoprotective effects of RSSH precursors against DOX-induced cardiotoxicity in H9c2 cardiomyoblasts. First, we assessed the cytotoxicity of the RSSH donors (**AST-2**, **APT-1**, **AST-2-TPP**, and **APT-1-TPP**) using the CCK-8 assay.^[24]^ All compounds are well tolerated by H9c2 cells, showing no measurable toxicity up to 200 μM (SI, Figure S23). To evaluate cytoprotection, H9c2 cells were pretreated with RSSH donors for 4 h, followed by 24 h co-treatment with DOX (5 μM). As expected, DOX alone reduced cell viability to ca. 50% (Figure 4a). Both **AST-2** and its mitochondria-targeted analogue **AST-2-TPP** conferred dose-dependent protection, with significant effects observed at ≥1 μM and maximal protection achieved at 25 μM (Figure 4a). Although both compounds were protective, no enhancement of potency was observed upon TPP conjugation. Furthermore, **APT-1** and **APT-1-TPP** exhibited a similar dose-dependent cytoprotective effect (Figure 4b). Under similar experimental conditions, the thiol controls *N*-acetyl penicillamine methyl ester (Pen-SH) and its TPP-conjugated analogue (TPP-SH, **5**) failed to mitigate DOX-induced cytotoxicity (Figure 4c), suggesting that the observed protection is likely attributed to RSSH or RSSH-derived species. A distinctive feature of our study is the direct comparison between mitochondria-targeted and non-targeted RSSH donors. In contrast to H2S donors, where mitochondrial targeting improves efficacy (e.g., the mitochondria-targeted donor AP39 is more effective than the untargeted donor ADT-OH),^[36,45,46]^ TPP conjugation only modestly enhances the cytoprotective effects of RSSH donors under our experimental conditions. This outcome may reflect the intrinsic high efficacy of non-targeted RSSH donors, thereby limiting the additional benefit of mitochondrial targeting.

### Mitochondria-Targeted Hydropersulfide Donor Preserves Doxorubicin Activity in Cancer Cells

**Figure 5.**
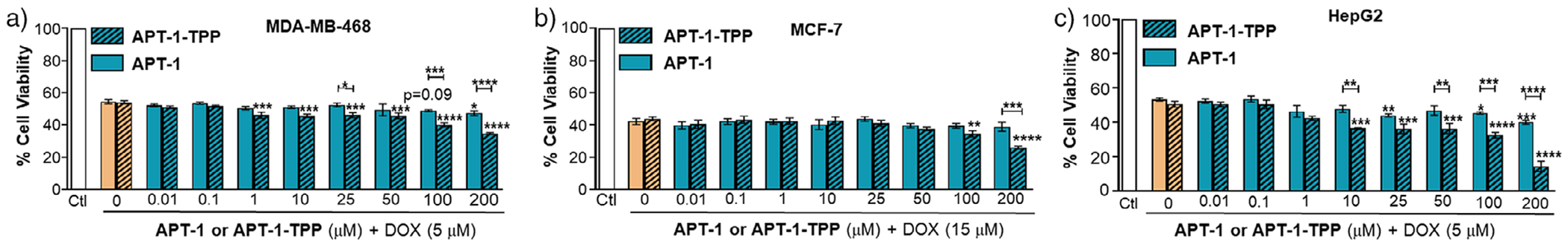
Effect of RSSH donors on anticancer activity of DOX in cancer cells. MDA-MB-468, MCF-7, and HepG2 cells were pretreated with **APT-1** or its mitochondria-targeted analog **APT-1-TPP** for 4 h, followed by co-treatment with DOX (5 μM for MDA-MB-468 and HepG2; 15 μM for MCF-7) for an additional 24 h. Results are presented as mean ± SEM, *n* = 3; *p < 0.05, **p < 0.01, ***p < 0.001, ****p < 0.0001. Ctl = vehicle-treated group (0.01% DMSO in medium).

We next evaluated the effects of RSSH donors on DOX activity in three representative cancer cell lines: triple-negative breast cancer MDA-MB-468, ER-positive breast cancer MCF-7, and hepatocellular carcinoma HepG2. Again, the **APT-1** / **APT-1-TPP** pair was selected for this study based on their extended half-lives, superior aqueous stability, and modestly improved performance in cardiomyoblast assays. In MDA-MB-468 cells, pretreatment with **APT-1** did not provide cytoprotection against DOX (Figure 5a). **APT-1-TPP** likewise showed no protective effect; instead, at concentrations ≥100 μM, it modestly enhanced DOX’s anticancer activity. A similar trend was observed in MCF-7 cells (Figure 5b). In HepG2 cells, **APT-1** again showed no protective effect, whereas **APT-1-TPP** potentiated DOX cytotoxicity in a dose-dependent manner (Figure 5c). Notably, both **APT-1** and **APT-1-TPP** are non-toxic to HepG2 cells when treated alone at concentrations up to 200 μM (Figure S23D). These findings demonstrate that the non-targeted donor **APT-1** neither protects nor augments DOX efficacy in cancer cells, whereas the mitochondria-targeted **APT-1-TPP** enhances DOX cytotoxicity.

### RSSH Donors Preserve Mitochondrial Membrane Potential/ATP Levels in Cardiomyocytes Exposed to DOX but Not in Cancer Cells

**Figure 6.**
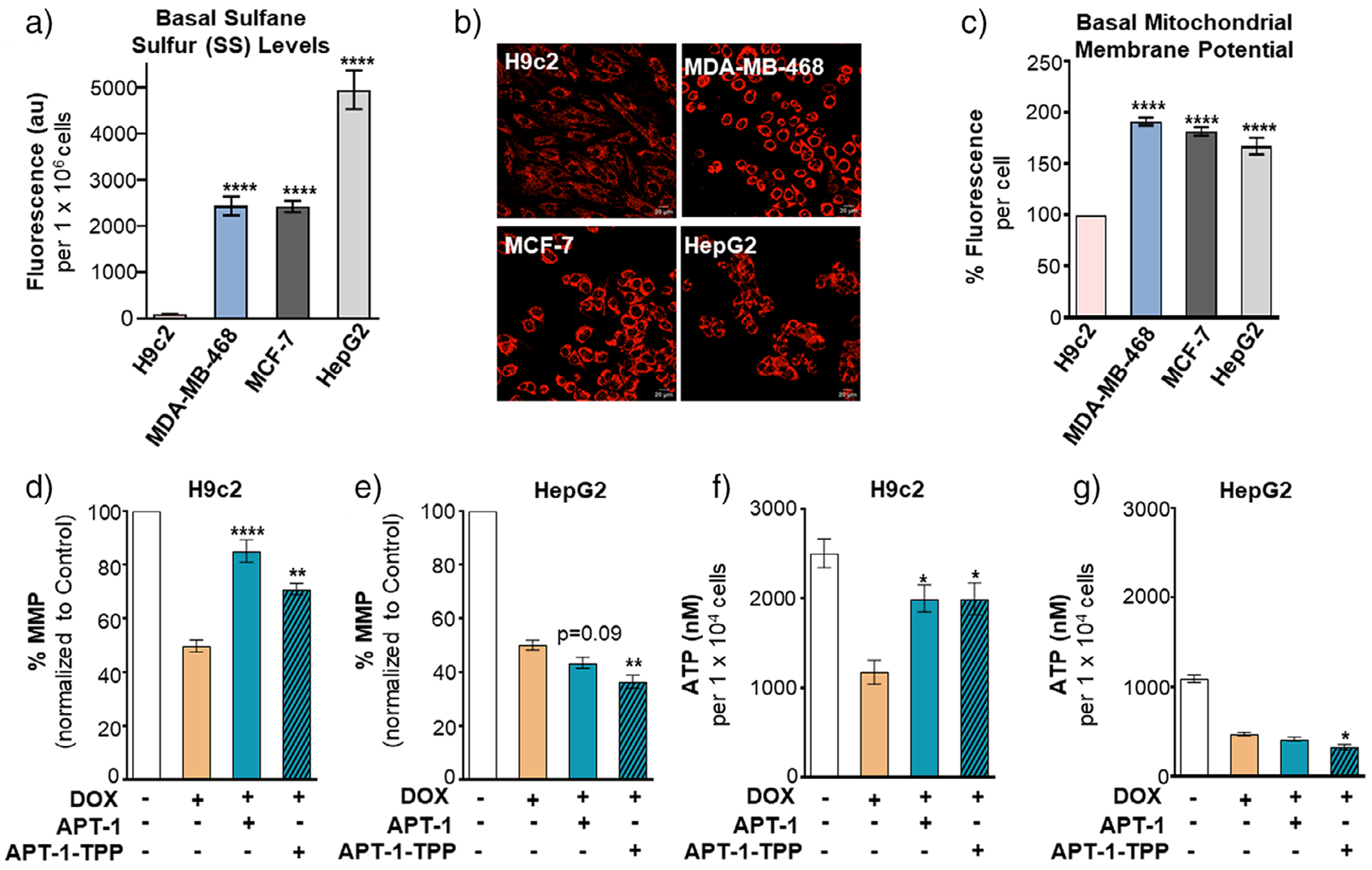
a) Basal sulfane sulfur (SS) levels in H9c2 cardiomyocyte, MDA-MB-468, MCF-7, and HepG2 cell lines as detected by SSP4 fluorescence. b) Representative images and c) basal levels of mitochondrial membrane potential (MMP) in H9c2 and cancer cells (MDA-MB-468, MCF-7, and HepG2). d) Impact of **APT-1** (25 μM) or **APT-1-TPP** (25 μM) on MMP in DOX (5 μM)-treated (d) H9c2 and e) HepG2 cells. Impact of **APT-1** (25 μM) and **APT-1-TPP** (25 μM) on ATP levels in DOX (5 μM)-treated f) H9c2 and g) HepG2 cells. Results are expressed as mean ± SEM, *n* = 3–6; *P < 0.05, **P < 0.01, ***P < 0.001, ****P < 0.0001. Significances in (a) and (c) are presented with respect to H9c2 cells.

Mechanistic studies were conducted to understand why RSSH donors protect cardiomyoblasts but preserve or enhance DOX’s activity in cancer cells. First, we measured endogenous sulfane sulfur (SS) levels using the fluorescent probe SSP4.^[22,47]^ All three cancer cell lines (MDA-MB-468, MCF-7, and HepG2) exhibited significantly higher basal SS levels relative to H9c2 cells, with HepG2 cells displaying the highest levels (Figure 6a). This result suggests sulfur metabolism in cancer cells is distinct from that in cardiac cells.^[48]^

Next, we measured basal mitochondrial membrane potential (MMP) using tetramethyl rhodamine methyl ester (TMRM) fluorescence.^[49]^ Cancer cells showed elevated MMP compared with H9c2 cells (Figures 6b and c). Although cancer cells rely more on the glycolytic system in the cytoplasm rather than oxidative phosphorylation in the mitochondria for ATP production, it is now accepted that cancer cells are metabolically flexible and use both glycolysis and oxidative phosphorylation to support proliferation and often require enhanced mitochondrial ATP production.^[50]^ It is also reported that higher ATP production helps cancer cells acquire drug resistance and metastatic potential^[51]^ Thus, our finding of high MMP in cancer cells aligns with the high metabolic activity of cancer cells, which rely on sustained MMP and redox capacity to support growth and survival.^[52,53]^

We then examined how RSSH donors influence MMP during DOX exposure. As expected, DOX alone induced mitochondrial depolarization in H9c2 cells, reducing MMP to ca. 50% of control (Figure 6d). Pre-treatment with **APT-1** preserved MMP, and **APT-1-TPP** also conferred protection, though to a slightly lesser extent. In contrast, neither donor prevented DOX-induced mitochondrial depolarization in HepG2 cells (Figure 6e). Notably, the combination of **APT-1-TPP** and DOX further exacerbated MMP loss compared with DOX alone, indicating a potential synergistic effect. To complement these observations, we quantified intracellular ATP levels as a functional readout of mitochondrial activity. In H9c2 cells, DOX treatment significantly depleted ATP, whereas pre-treatment with either **APT-1** or **APT-1-TPP** restored DOX-mediated reduced ATP levels (Figure 6f). In HepG2 cells, however, neither donor rescued DOX-induced ATP depletion (Figure 6g), reinforcing the lack of mitochondrial protection in cancer cells.

The observed cell-type-specific effects of RSSH donors likely stem from intrinsic differences in basal sulfane sulfur levels, mitochondrial physiology, and redox buffering capacity between H9c2 cardiac cells and cancer cells. H9c2 cells, which possess high mitochondrial content, respond to RSSH donors by attenuating DOX-induced loss of MMP and ATP depletion (Figure 6d–f). Mechanistically, these cardioprotective effects may arise from activation of Nrf2-dependent antioxidant pathways and enhanced mitochondrial biogenesis via PGC-1*α*, as demonstrated in our previous work.^[22]^

In contrast, HepG2 cells, which rely heavily on ATP production to sustain proliferation and metastasis, exhibit a different response. RSSH donor treatment disrupts their redox balance, and consequently DOX-induced reductions in MMP and ATP are not rescued, and in some cases are further exacerbated. This observation aligns with current anticancer strategies that seek to impair mitochondrial function and deplete ATP to inhibit cancer cell growth. Moreover, because HepG2 cells maintain elevated basal SS levels and high MMP, additional RSSH appears to exceed their redox buffering capacity, potentially triggering reductive stress and increasing their susceptibility to DOX-induced mitochondrial dysfunction. Taken together, these findings indicate that exogenous RSSH supplementation protects cardiac cells by mitigating DOX-induced oxidative stress and preserving mitochondrial function, while simultaneously compromising mitochondrial homeostasis in cancer cells.

## Conclusion

Developing new therapeutic agents that protect the heart from anthracycline-induced toxicity without compromising anticancer activity remains a critical challenge in cardiooncology. Our work shows that 1) esterase-sensitive RSSH donors protect H9c2 cardiomyocytes against DOX toxicity; 2) mitochondria-targeted RSSH donors enhance the anticancer activity of DOX in several cancer cell lines; and 3) RSSH supplementation prevents DOX-induced mitochondrial injury in cardiomyocytes but not in hepatocellular carcinoma cells.

A distinguishing feature of these donors is the release of tertiary alkyl hydropersulfides, which enables efficient sulfane sulfur transfer to biological thiols, producing corresponding hydropersulfides (e.g., Cys-SSH and GSSH). Furthermore, these precursors exhibit improved aqueous stability and efficient RSSH release with tunable kinetics. **AST-2** liberates RSSH directly upon enzymatic hydrolysis, while **APT-1** undergoes sequential deacetylation and intramolecular cyclization, enabling more gradual release. Conjugation with a triphenylphosphonium moiety maintains enzymatic activation while directing RSSH to mitochondria. In cell-based LC/MS studies, untargeted donors increased levels of cytosolic RSSH (along with RSSH-derived species, H_2_S_2_ and H_2_S), while TPP-conjugated analogues selectively elevated mitochondrial levels. Both donor classes protected cardiomyocytes from DOX toxicity, with no added benefit from mitochondria-targeted donors, likely because untargeted compounds already achieved near-maximal effects at low concentrations. In cancer cells, however, mitochondria-targeted **APT-1-TPP** did not confer protection; instead, it showed synergy with DOX-induced cell toxicity. These contrasting outcomes likely reflect differences in redox status and mitochondrial physiology: cardiomyocytes, with low basal sulfane sulfur levels, are protected against DOX by RSSH supplementation, whereas cancer cells, with higher basal sulfane sulfur levels and elevated MMP, accumulate RSSH to harmful levels. Overall, our work presents new mitochondria-targeted RSSH donors as promising cardioprotective agents and as chemical biology tools to explore the emerging role of RSSH signaling in mitochondrial function.

## Supplementary Material

Supporting Info

Experimental procedures and Supporting Information Figures S1–S32. The authors have cited additional references within the Supporting Information.^[22–24, 54–58]^

Additional supporting information can be found online in the Supporting Information section

## Figures and Tables

**Table 1:** Hydropersulfide yields and kinetic parameters of RSSH donors.

Precursor	RSSH yield (%)^a)^	*t*_1/2_ (min) Precursor consumption	*t*_1/2_ (min) Intermediate formation	*t*_1/2_ (min) Intermediate decay	*t*_1/2_ (min) RSSH release
**AST-2**	95	50.1 ± 1.8	NA	NA	48.3 ± 2.1
**ApT-1**	92	17.1 ± 0.4	14.8 ± 1.5	96.9 ± 6.3	104.9 ± 9.8
**AsT-2-TPP**	91^b)^	20.7 ± 0.6	NA	NA	20.1 ±1.6
**ApT-1-TPP**	95	5.2 ± 0.2	4.7 ± 0.1	142.5 ± 7.0	124.8 ± 9.4

a) RSSH precursors (25 μM) were incubated in the presence of HPE-IAM (250 μM) in pH 7.4 ammonium bicarbonate containing DTPA at 37 °C.

b) Given the shorter half-life of **AST-2-TPP**, RSSH trapping was performed using 50 equiv of HPE-IAM to improve trapping efficiency. Reported data represent mean ± SD, *n* = 3.

## Data Availability

The data that support the findings of this study are available from the corresponding author upon reasonable request.
