# Supplementary material for: Esterase-Responsive Mitochondria-Targeted Hydropersulfide Donors Mitigate Doxorubicin Cardiotoxicity While Preserving Anticancer Activity
Source: Angew Chem Int Ed Engl. Author manuscript; Available in PMC 2026 Jun 1. (PMC13224100; doi:10.1002/anie.202521645)
Supplement: Supporting Info [file NIHMS2174897-supplement-Supporting_Info.pdf]

Esterase-Responsive Mitochondria-Targeted Hydropersulfide Donors Mitigate Doxorubicin Cardiotoxicity While Preserving Anticancer Activity

Jinjing Gu,[a] Qi Liu,[a] Deborah Rodriguez,[a] Jordan Lamar,[a] Klaire R. Bradley,[a] Gizem Keceli,[b] Andrew Thampoe,[a] Yihang Xiao,[a] Nazareno Paolocci,[b,c] Vinayak S. Khodade,*[a,d] John P. Toscano*[a]

[a] *Department of Chemistry, Johns Hopkins University, Baltimore, MD, 21218, USA*
[b] *Division of Cardiology, Johns Hopkins University School of Medicine, Baltimore, Maryland 21205, United States*
[c] *Department of Biomedical Sciences, University of Padova, Padova, Italy*
[d] *Smidt Heart Institute, Department of Cardiac Surgery, Cedars-Sinai Medical Center, Los Angeles, CA, 90048, USA*

Table of Contents

General Information	S2
Synthesis and Characterization	S2 – S6
RSSH Generation Studies by HPLC and LC-MS	S6 – S16
RSSH Precursors Stability with Glutathione	S16 – S17
Intracellular RSSH analysis by LC-MS/MS	S17 – S19
Cell Viability Study	S19 – S20
Sulfane Sulfur Measurement	S20 – S21
Mitochondrial Membrane Potential Measurement	S21
ATP Production Measurement	S22
References	S22
NMR Spectra	S23 – S30

General Information

Starting materials, reagents, and solvents were purchased from commercial suppliers (Sigma-Aldrich, Oakwood Chemical, and TCI) and used without further purification unless otherwise noted. Deuterated solvents were obtained from Cambridge Isotope Laboratories. Analytical thin-layer chromatography (TLC) was performed on silica gel 60 F254 aluminum-backed plates (Merck/Sigma-Aldrich) and visualized under UV light at 254 nm. Majority of NMR spectra were recorded on a Bruker Avance III 400 MHz spectrometer. A few were recorded on a Bruker Avance III (600 MHz) and a Bruker Avance NEO (800 MHz), equipped with TCI cryogenic probes, optimized for high-sensitivity ^{1}H and ^{13}C detection. Chemical shifts (δ) are reported in parts per million (ppm) relative to residual solvent peaks as internal references ($CDCl_3$: ^{1}H δ = 7.26 ppm, ^{13}C δ = 77.16 ppm; DMSO-d_6: ^{1}H δ = 2.50 ppm, ^{13}C δ = 39.52 ppm). High-resolution mass spectra (HRMS) were obtained on a Waters ACQUITY UPLC system equipped with a Xevo G2-S QToF mass spectrometer using electrospray ionization (ESI). The kinetics of hydropersulfide (RSSH) release were monitored by UPLC–MS and HPLC. UPLC–MS analyses were performed on a Waters ACQUITY UPLC system equipped with a Xevo G2 QToF detector and an ACQUITY UPLC BEH C18 column (2.1 × 50 mm, 1.7 μm). HPLC analyses were carried out on an Agilent 1260 Infinity II system with UV detection using a Phenomenex Luna C18 reversed-phase column (4.6 × 150 mm, 5 μm). RSSH levels in cytoplasmic and mitochondrial fractions were measured on an Agilent Ultivo triple quadrupole LC–MS system equipped with a reversed-phase column (Agilent Poroshell 120, EC-C18 (4.6 x 100 mm, 2.7 μm). pH measurements were obtained using a Fisher Scientific Accumet AB15 pH meter.

Synthesis and Characterization:

A. Synthesis of Hydropersulfide Donors

Methyl 2-acetamido-3-((methoxycarbonyl)disulfaneyl)-3-methylbutanoate (AST-2)

Scheme S1

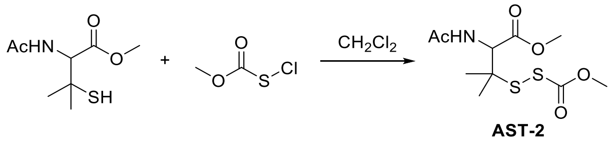

To a 100 mL round-bottom flask, *N*-acetyl penicillamine methyl ester (410 mg, 2 mmol) was dissolved in anhydrous dichloromethane (10 mL), and the solution was cooled to 0 °C under a nitrogen atmosphere. A solution of methoxycarbonylsulfenyl chloride (278 mg, 2.1 mmol) in anhydrous dichloromethane (5 mL) was then added dropwise at 0 °C. The reaction mixture was stirred at 0 °C for 1 h. The volatiles were removed under reduced pressure, and the residue was purified by flash column chromatography on silica gel using hexane/ethyl acetate as eluents to afford **AST-2** as a colorless semisolid (422 mg, 72% yield). ^{1}H NMR (400 MHz, DMSO-d_6) δ 8.37 (d, *J* = 9.0 Hz, 1H), 4.58 (d, *J* = 9.0 Hz, 1H), 3.85 (s, 3H), 3.65 (s, 3H), 1.90 (s, 3H), 1.32 (s, 3H), 1.31 (s, 3H); ^{13}C NMR (75 MHz, DMSO-d_6) δ 170.51, 170.09, 169.16, 58.09, 56.49, 53.05, 52.45, 24.85, 23.65, 22.67; HRMS (ESI) *m/z* [M + Na]$^{+}$ calcd for $C_{10}H_{17}NNaO_5S_2^{+}$ $C_{10}H_{17}NNaO_5S_2^{+}$: 318.0440; found: 318.0456.

Methyl 2-acetamido-3-(((2-acetoxyethyl)(methyl)carbamoyl)disulfaneyl)-3-methylbutanoate (APT-1)

Scheme S2

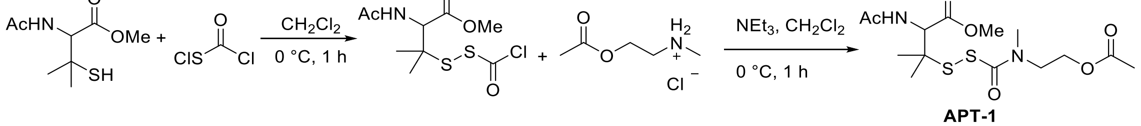

To a solution of *N*-acetyl penicillamine methyl ester (410 mg, 2.0 mmol) in anhydrous dichloromethane (10 mL), a solution of chlorocarbonylsulfenyl chloride (314 mg, 2.4 mmol) in anhydrous dichloromethane (5 mL) was added dropwise at 0 °C under a nitrogen atmosphere. The mixture was stirred at 0 °C for 1 h, then volatiles were removed under reduced pressure to obtain methyl-2-acetamido-3-((chlorocarbonyl)disulfanyl)-3-methylbutanoate intermediate, which was used for the next step without further purification. This intermediate was redissolved in dichloromethane (5 mL) and cooled to 0 °C. In a separate flask, 2-acetoxy-*N*-methylethan-1-aminium chloride[1] (399 mg, 2.6 mmol) was neutralized using triethylamine (405 mg, 4.0 mmol) in dichloromethane (10 mL), and this solution was added to the reaction mixture. The resulting mixture was stirred at 0 °C for 1 h. The mixture was quenched with water and extracted with dichloromethane (20 mL × 3). The combined organic layers were washed with brine, dried over sodium sulfate, and concentrated under vacuum. The residue was purified by flash column chromatography on silica gel using hexane/ethyl acetate as eluents to afford the **APT-1** as a colorless semisolid (711 mg, 93% yield). ^{1}H NMR (400 MHz, $CDCl_3$) δ 7.54 (bs, 1H), 4.54 (d, *J* = 8.3 Hz, 1H), 4.23 (bs, 2H, rotamers), 3.72 (s, 3H), 3.71 – 3.66 (m, 2H), 3.12 (s, 3H, rotamers), 2.05 (s, 6H), 1.46 (s, 3H), 1.33 (s, 3H); ^{13}C NMR (101 MHz, $CDCl_3$) δ 170.7, 170.6, 170.5, 167.7, 61.6, 61.1, 59.3, 52.7, 52.2, 49.7, 49.1, 36.7, 36.3, 26.6, 26.1, 23.0, 20.9; HRMS (ESI) *m/z* [M + H]$^+$ calcd for $C_{14}H_{25}N_2O_6S_2^+$: 381.1149; found: 381.1153.

Note: The signal at δ 4.23 ppm (2H) corresponds to a methylene group expected to appear as a triplet but is observed as a broad singlet due to poor resolution of rotamers. The *N*-methyl at δ 3.12 ppm is expected to be a singlet but appears as two singlets (integrating to a total of 3H), also attributed to rotamers. Variable-temperature NMR experiments were attempted to resolve the rotamers; however, **APT-1** decomposed at elevated temperatures (80 °C) in DMSO-d_6.

[3-(Methylamino)propyl]triphenylphosphonium dibromide (2)

In a two-necked round-bottom flask, 3-bromo-*N*-methylpropanamine hydrobromide[2] (8.9 g, 38.2 mmol) and triphenylphosphine (10.02 g, 38.2 mmol) were dissolved in anhydrous acetonitrile (100 mL) under a nitrogen atmosphere. The reaction mixture was refluxed for 96 h, during which a white precipitate formed. The precipitate was filtered, washed thoroughly with acetonitrile and diethyl ether, and dried to obtain the desired product as a white solid (15.2 g, 81%). ^{1}H NMR (400 MHz, DMSO-d_6) δ 8.74 (bs, 2H), 7.95 – 7.75 (m, 15H), 3.9 - 3.82 (m, 2H), 3.12 (t, *J* = 7.1 Hz, 2H), 2.53 (s, 3H), 1.97 -1.91 (m, 2H); ^{13}C NMR (101 MHz, DMSO-d_6) δ 135.1, 133.7, 133.6, 130.4, 130.3, 118.4, 117.5, 48.1, 47.9, 32.3, 18.7, 18.1; HRMS (ESI) *m/z* [M + H]$^+$ calcd for $C_{22}H_{25}N_2P^+$: 334.1720; found: 334.1723.

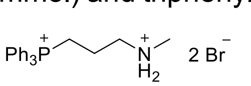

(3-(2-Acetamido-3-mercapto-N,3-dimethylbutanamido)propyl)triphenylphosphonium bromide (4)

[3-(Methylamino)propyl]triphenylphosphonium bromide, hydrobromide (988 mg, 2.0 mmol), and thiolactone **3**[3] (381 mg, 2.2 mmol) were dissolved in anhydrous dichloromethane (20 mL) and stirred under a nitrogen atmosphere. To this heterogeneous solution, *N*,*N*-diisopropylethylamine (DIPEA, 258 mg, 2.0 mmol) was added dropwise at room temperature. The reaction mixture was stirred at room temperature for 2 h, then diluted with water (30 mL) and extracted with dichloromethane (3 × 30 mL). The combined organic layers were washed with brine, dried over anhydrous sodium sulfate, filtered, and concentrated under reduced pressure to afford a colorless semisolid. The crude product was purified by flash column chromatography on silica gel using dichloromethane/methanol as the eluent, followed by reversed-phase chromatography on a C18 column using an acetonitrile/water mobile phase to obtain the desired product **4** as a white solid (753 mg, 64%). ^{1}H NMR (400 MHz, $CDCl_3$) δ 7.87–7.65 (m, 15H), 6.76 (d, *J* = 8.4 Hz, 0.66H, rotamer A), 6.49 (d, *J* = 9.0 Hz, 0.34H, rotamer B), 4.94 (d, *J* = 9.0 Hz, 0.34H, rotamer B), 4.77 (d, *J* = 8.5 Hz, 0.66H, rotamer A), 4.28–

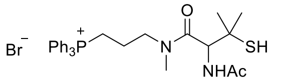

4.22 (m, 0.7H), 3.98–3.84 (m, 2.6H), 3.71–3.65 (m, 0.7H), 3.32 (s, 2H, rotamer A), 2.94 (s, 1H, rotamer B), 2.72 (s, 0.35H, rotamer B), 2.61 (s, 0.64H, rotamer A), 1.97–1.83 (m, 5H), 1.39 (s, 1.9H), 1.36 (s, 1.1H), 1.33 (s, 3H); ^{13}C NMR (101 MHz, $CDCl_3$) δ 171.3, 170.5, 170.5, 169.7, 135.2, 135.1, 135.1, 135.0, 133.8, 133.7, 133.7, 133.6, 118.4, 118.4, 117.6, 117.5, 55.6, 54.5, 50.2, 48.3, 48.1, 46.8, 46.2, 37.3, 34.1, 30.7, 30.3, 28.6, 28.6, 23.2, 23.0, 21.9, 20.4, 20.2, 19.9, 19.7; HRMS (ESI) *m/z* [M]$^+$ calcd for $C_{29}H_{36}N_2O_2PS^+$: 507.2230; found: 507.2234.

Note: Compounds **4**, **AST-2-TPP, APT-1-TPP, S1, and TPP-RSS-HPE-AM** exist as rotameric mixtures in solution. Integration discrepancies in the ^{1}H NMR spectra and signal duplication in ^{1}H and ^{13}C NMR spectra are observed for these compounds.

(3-(2-Acetamido-3-((methoxycarbonyl)disulfaneyl)-N,3-dimethylbutanamido)propyl)triphenylphosphonium bromide (AST-2-TPP)

To a 100 mL round-bottom flask, TPP-*N*-acetylpenicillamine **4** (300 mg, 0.51 mmol) was dissolved in anhydrous dichloromethane (5 mL), and the solution was cooled to 0 °C. A solution of methoxycarbonylsulfenyl chloride (**5**) (71 mg, 0.56 mmol) in anhydrous dichloromethane (3 mL)

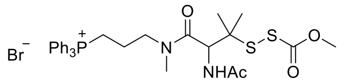

was added dropwise at 0 °C under a nitrogen atmosphere. The resulting reaction mixture was stirred at 0 °C for 1 h. Volatiles were removed under reduced pressure, and the residue was washed with diethyl ether. The crude product was purified by reverse-phase C18 chromatography using acetonitrile as the mobile phase to afford **AST-2-TPP** as a white solid (148 mg, 43% yield). ^{1}H NMR (400 MHz, DMSO-d_6) δ 8.33 (d, J = 9.0 Hz, 1H), 7.93–7.89 (m, 3H), 7.84–7.74 (m, 12H), 5.13 (d, J = 9.0 Hz, 0.35H, rotamer B), 4.94 (d, J = 9.0 Hz, 0.66H, rotamer A), 3.83 (s, 1H, rotamer B), 3.81 (s, 2H, rotamer A), 3.66–3.40 (m, 4H), 3.02 (s, 2H, rotamer A), 2.77 (s, 1H, rotamer B), 1.86–1.71 (m, 5H), 1.50–1.18 (4 × s, 6H); ^{13}C NMR (151 MHz, $CDCl_3$) δ 171.1, 171.0, 170.9, 170.8, 170.7, 170.6, 170.5, 170.1, 169.9, 169.8, 169.7, 135.2, 135.2, 135.0, 133.9, 133.8, 133.8, 133.7, 130.7, 130.6, 130.5, 130.4, 118.4, 118.3, 117.8, 117.7, 55.8, 55.7, 54.5, 54.4, 54.3, 54.1, 54.0, 53.9, 53.7, 53.2, 52.4, 50.4, 50.3, 48.6, 48.5, 48.4, 48.3, 37.5, 37.3, 36.9, 34.4, 34.4, 25.6, 25.5, 25.3, 25.2, 25.1, 25.0, 25.0, 24.9, 24.8, 24.5, 23.7, 23.3, 23.2, 23.1, 23.0, 22.8, 22.3, 22.2, 20.5, 20.4, 20.3, 20.1, 20.0; HRMS (ESI) *m/z* [M]$^+$ calcd for $C_{31}H_{38}N_2O_4PS_2^+$: 597.2005; found: 597.2013.

(11-acetamido-6,10,10,13-tetramethyl-2,7,12-trioxo-3-oxa-8,9-dithia-6,13-diazahexadecan-16-yl)triphenylphosphonium bromide (APT-1-TPP)

To a solution of 2-acetoxy-*N*-methylethan-1-aminium trifluoroacetate salt (137 mg, 0.59 mmol) in

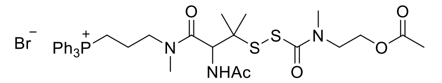

dichloromethane (15 mL), a solution of chlorocarbonylsulfenyl chloride (69 mg, 0.53 mmol) in dichloromethane (5 mL) was added at 0 °C under an inert atmosphere, followed by the addition of triethylamine (54 mg, 0.53 mmol). The resulting mixture was stirred at 0 °C for 1 h to produce sulfenyl chloride intermediate **6** *in situ*. To the same reaction mixture, a solution of TPP-*N*-acetylpenicillamine **4** (100 mg, 0.20 mmol) in dichloromethane (5 mL) was added under an inert atmosphere. The reaction was stirred at room temperature for 1 h. Upon completion (by TLC analysis), the volatiles were removed under reduced pressure. The crude product was washed with anhydrous diethyl ether and hexanes, and purified by reverse-phase HPLC using a C18 column with water and acetonitrile as eluents. The desired product **APT-1-TPP** was obtained as a white solid after lyophilization (50 mg, 33% yield). ^{1}H NMR (600 MHz, $CDCl_3$) δ 7.83 – 7.63 (m, 15H), 7.59 (bs, 0.72H), 7.41(d, J = 8.0 Hz, 0.72H), 4.99 (d, J = 9.1 Hz, 0.28H), 4.74 (d, J = 7.2 Hz, 0.72H), 4.20 (bs, 2H), 3.98 – 3.90 (m, 1H), 3.70 – 3.60 (m, 4H), 3.48 – 3.44 (m, 1H), 3.26 (s, 2H), 3.16 (s, 2H), 3.05 (s, 1H), 2.90 (s, 1H), 2.04 (s, 3H), 2.00 – 1.89 (m, 5H), 1.38 (s, 2.1H), 1.36 (s, 0.9H), 1.31 (s, 2.1H), 1.24 (s, 0.9H); ^{13}C NMR (151 MHz, $CDCl_3$) δ 170.7, 170.5, 170.5, 169.9, 169.1, 167.8, 167.4, 135.1, 135.0, 134.9, 134.0, 133.9, 133.8, 133.7, 133.7, 133.6, 130.6, 130.5, 118.7, 118.6, 118.5, 118.1, 118.1, 118.0, 61.7, 61.1, 55.1, 54.4, 53.8, 53.2, 50.3, 50.2, 49.6, 49.0, 48.6, 48.5, 37.5, 36.6, 36.3, 34.3, 30.2, 26.4,

26.2, 26.0, 25.6, 25.5, 25.4, 25.2, 22.9, 22.9, 22.5, 21.7, 20.9, 20.6, 20.0, 19.6; HRMS (ESI) *m/z* $[M]^+$ calcd for $C_{35}H_{45}N_3O_5PS_2^+$: 682.2533; found: 682.2530.

B. Synthesis of Authentic Standards (RSS-HPE-AM and TPP-RSS-HPE-AM)

Authentic **RSS-HPE-AM** was synthesized following our previously reported method.[4]

(3-(2-acetamido-3-((2-((4-hydroxyphenethyl)amino)-2-oxoethyl)disulfaneyl)-N,3-dimethylbutanamido)propyl)triphenylphosphonium (TPP-RSS-HPE-AM)

Scheme S3

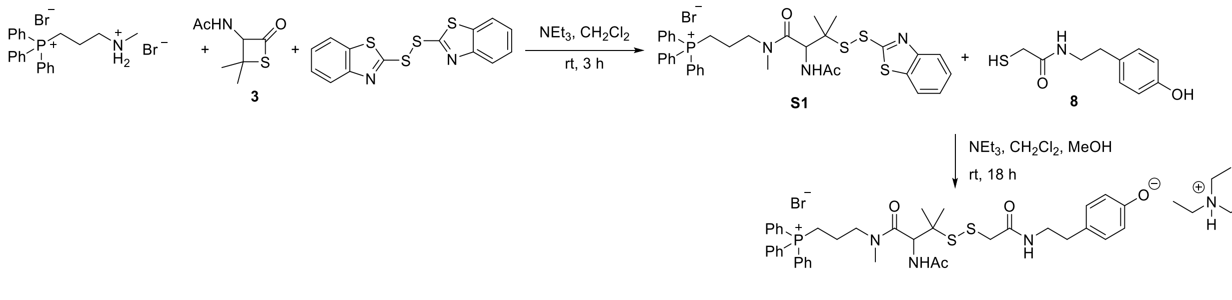

(3-(2-acetamido-3-(benzo[d]thiazol-2-yldisulfaneyl)-N,3 dimethylbutanamido)propyl)triphenylphosphonium bromide (Compound S1)

To a solution of [3-(Methylamino)propyl]triphenylphosphonium bromide, hydrobromide (494 mg, 1.0 mmol), thiolactone **3** (173 mg, 1.0 mmol), and 1,2-bis(benzo[d]thiazol-2-yl)disulfane (366 mg, 1.1 mmol) in anhydrous dichloromethane (20 mL) under a nitrogen atmosphere, triethylamine (152 mg, 1.5 mmol) was added dropwise, and the reaction mixture was stirred at room temperature for 16 h. Upon completion of reaction, mixture was diluted with water (30 mL) and extracted with dichloromethane (3 × 30 mL). The combined organic layers were dried over anhydrous sodium sulfate, filtered, and concentrated under reduced pressure to afford a light yellow solid. The crude product was purified by flash column chromatography on silica gel using dichloromethane/methanol as the eluent to obtain the desired product compound **S1** as light yellow solid (504 mg, 67%). ^{1}H NMR (800 MHz, DMSO-d_6) δ 8.45 (d, J = 9.6 Hz, 0.34H, rotamer A), 8.42 (d, J = 8.9 Hz, 0.66H, rotamer B), 8.06 – 7.75 (m, 17H), 7.51 – 7.41 (m, 2H), 5.25 (d, J = 9.6 Hz, 0.34H, rotamer A), 5.09 (d, J = 8.9 Hz, 0.66H, rotamer B), 3.87 – 3.42 (m, 4H), 3.05 (s, 2H, rotamer B), 2.80 (s, 1H, rotamer A), 1.86 (s, 1H, rotamer A), 1.85 (s, 2H, rotamer B), 1.84 – 1.72 (m, 2H), 1.45 (s, 3H), 1.39 (s, 2H, rotamer B), 1.38 (s, 1H, rotamer A); ^{13}C NMR (201.2 MHz, DMSO-d_6) δ 173.4, 173.2, 170.2, 170.1, 169.8, 169.2, 154.8, 135.7, 135.5, 134.1, 134.1, 134.0, 130.8, 130.7, 127.1, 125.4, 122.4, 122.2, 122.2, 119.0, 118.5, 56.6, 56.3, 55.4, 53.4, 52.5, 50.1, 50.0, 48.0, 47.9, 36.4, 33.9, 24.9, 24.8, 24.2, 22.7, 22.6, 21.8, 20.2, 18.8, 18.7, 18.5, 18.5. MS (MS-ESI) *m/z* $[M]^+$ calcd for $C_{36}H_{39}N_3O_2PS_3^+$: 672.19; found: 672.13.

(3-(2-acetamido-3-((2-((4-hydroxyphenethyl)amino)-2-oxoethyl)disulfaneyl)-N,3-dimethylbutanamido)propyl)triphenylphosphonium (TPP-RSS-HPE-AM)

To a solution of compound **S1** (230 mg, 0.342 mM and (4-hydroxyphenethyl)-2-mercaptoacetamide **8**[4] (72 mg, 0.342 mM) in anhydrous dichloromethane (20 mL) with a few drops of anhydrous methanol, triethylamine (73 mg, 0.718 mM) was added dropwise, and the reaction mixture was stirred at room temperature for 18 h. Upon completion of the reaction, it was concentrated under reduced pressure to afford a light yellow semisolid. The crude product was purified by flash column chromatography on silica gel using dichloromethane/methanol as the eluent to obtain the desired product **TPP-RSS-HPE-AM** (125 mg, 41%) as white solid. ^{1}H NMR (800 MHz, DMSO-d_6) δ 9.34 (bs, 1H), 9.16 (s, 1H), 8.22 – 8.20 (m, 1H), 8.18 – 8.14 (m, 1H), 7.91 – 7.74 (m, 16H), 6.97 (d, J = 8.4 Hz, 1.32H, Rotamer A), 6.93 (d, J = 8.6 Hz, 0.65H, rotamer B),

6.67 (m, 2H), 5.20 (d, J = 9.5 Hz, 0.34H, rotamer B), 4.97 (d, J = 8.6 Hz, 0.66H, rotamer A), 3.96 (m, 0.34H, rotamer B), 3.61 (m, 0.66H, rotamer A), 3.50 – 3.46 (m, 2H,), 3.44 – 3.37 (m, 2H), 3.20 – 3.13 (m, 2H,), 3.12 – 3.08 (m, 6H)3.07 (s, 2H,), 2.76 (s, 1H), 2.56 (t, J = 7.4 Hz, 1.34H, rotamer A), 2.53 (t, J = 7.4 Hz, 0.66H, rotamer B), 1.83 (s, 1H), 1.80 (s, 2H), 1.76 – 1.72 (m, 1H), 1.31 – 1.30 (2 × s, 6H), 1.20 (t, 9H) ^{13}C NMR (201.2 MHz, DMSO-d_6) δ 170.1, 169.6, 169.5, 169.2, 167.3, 167.3, 155.7, 135.0, 134.9, 133.6, 133.6, 133.5, 130.3, 130.3, 130.2, 129.4, 129.3, 129.2, 118.6, 118.5, 118.1, 118.0, 115.1, 54.9, 53.5, 52.9, 52.4, 51.4, 49.7, 49.6, 47.2, 47.1, 45.6, 44.3, 44.3, 40.9, 40.8, 35.9, 34.2, 34.1, 33.2, 24.6, 24.1, 24.0, 23.7, 22.3, 22.1, 21.1, 19.7, 18.3, 18.1, 18.1, 17.9, 8.5; HRMS (ESI) m/z $[M]^+$ calcd for $C_{39}H_{47}N_3O_4PS_2^+$: 716.2741; found: 716.2723.

RSSH Generation Studies

A. Stability of RSSH Precursors.

Stock solutions of RSSH precursor (10 mM in DMSO) and HPE-IAM (100 mM in DMSO) were prepared immediately prior to the assay. The RSSH precursor (100 µM) was incubated in phosphate-buffered saline (PBS, pH 7.4) containing HPE-IAM (2 mM) at 37 °C. At designated time points, 200 µL aliquots were withdrawn and immediately quenched by mixing with an equal volume of ice-cold 0.1% formic acid. Samples were analyzed by Agilent HPLC with UV detection using a C18 reverse-phase column (Phenomenex Luna C18, 4.6 × 150 mm, 5 µm) at 25 °C with a flow rate of 1.0 mL/min and an injection volume of 20 µL. The gradient elution (all solvents v/v, containing 0.1% trifluoroacetic acid) was as follows: 0–10 min, linear gradient from 90% water / 10% acetonitrile to 50% water / 50% acetonitrile; 10–18 min, to 10% water / 90% acetonitrile; 18–20 min, held at 10% water / 90% acetonitrile; 20–25 min, return to 90% water / 10% acetonitrile. Absorbance was monitored at 275 nm. The percentage of precursor remaining at each time point was calculated by comparing the absorbance at 275 nm to the initial value. All experiments were performed in triplicate (n = 3), and data are reported as mean ± standard deviation (SD).

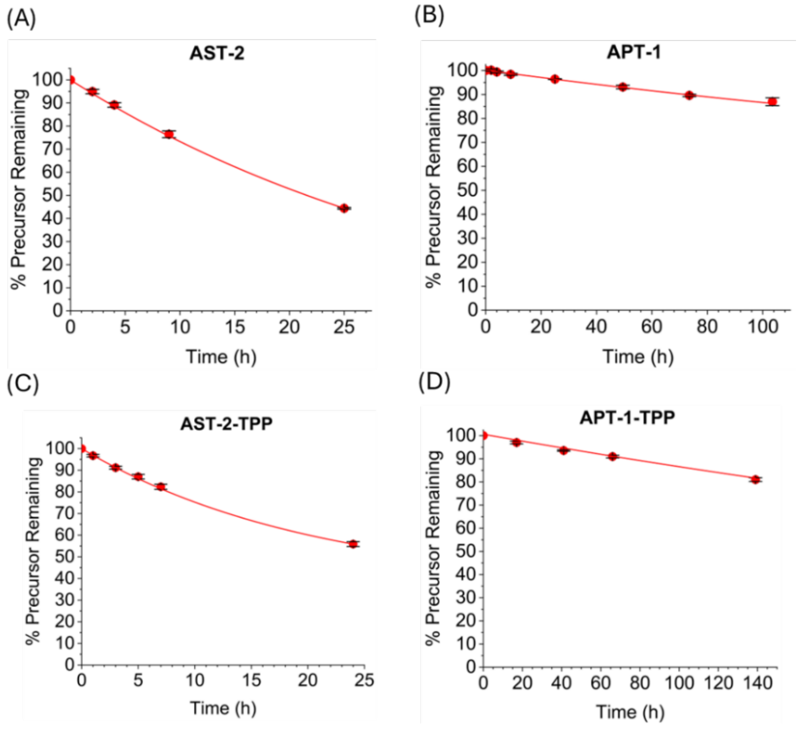

Figure S1. Stability profiles of (A) **AST-2**, (B) **APT-1**, (C) **AST-2-TPP**, and (D) **APT-1-TPP** in PBS, pH 7.4 at 37 °C in the absence of porcine liver esterase (PLE). Data are mean ± SEM (n = 3).

B. Kinetics of RSSH Generation.

Kinetics of RSSH generation were conducted as previously described.[5] Stock solutions of RSSH precursor (10 mM in DMSO), HPE-IAM (100mM in DMSO), and porcine liver esterase (PLE, 2000 U/mL in ammonium bicarbonate buffer) were freshly prepared. RSSH precursors (25 µM) were then immediately incubated with HPE-IAM (250 µM) and PLE (1 U/mL, from Sigma-Aldrich, E3019-20KU, ≥ 15 U/mg protein) in ammonium bicarbonate buffer (100 mM, pH 7.4) containing diethylenetriaminepentaacetic acid (DTPA, 100 µM) at 37 °C. At the indicated time points, 500 µL aliquots were withdrawn, quenched with concentrated formic acid (5 µL, 25.9 µM), and ultracentrifuged at 12,500 rpm for 5 min. The resulting supernatants were diluted with acetonitrile (200 µL supernatant + 300 µL of acetonitrile) and analyzed by UPLC–MS using a Waters Acquity H-Class system coupled to a Xevo G2 Q-Tof mass spectrometer operating in positive ion mode (MSe, 10 scans/s). Chromatographic separation was performed on a C18 reverse-phase column (Waters Acquity BEH C18, 2.1 × 50 mm, 1.7 µm) at 4 °C, with a flow rate of 0.3 mL/min and the following gradient (v/v; water containing 0.1% formic acid as solvent A, acetonitrile as solvent B): 0–1 min, 100% A; 1–7.5 min, linear gradient to 20% A / 80% B; 7.5–8.5 min, held at 20% A / 80% B; 8.5–10 min, return to 100% A. The injection volume was 5 µL. Mass spectral data were processed using MassLynx 4.1, and extracted ion chromatograms (EICs) corresponding to the exact masses of the RSSH precursor, intermediate, and the trapped product (RSS–HPE-AM) were integrated to quantify substrate consumption and product formation. Kinetic data were fitted to a single exponential curve using Origin. All experiments were performed in triplicate (n = 3), and results are reported as mean ± standard deviation (SD).

HRMS Data for Compounds Formed during RSSH Generation from AST-2

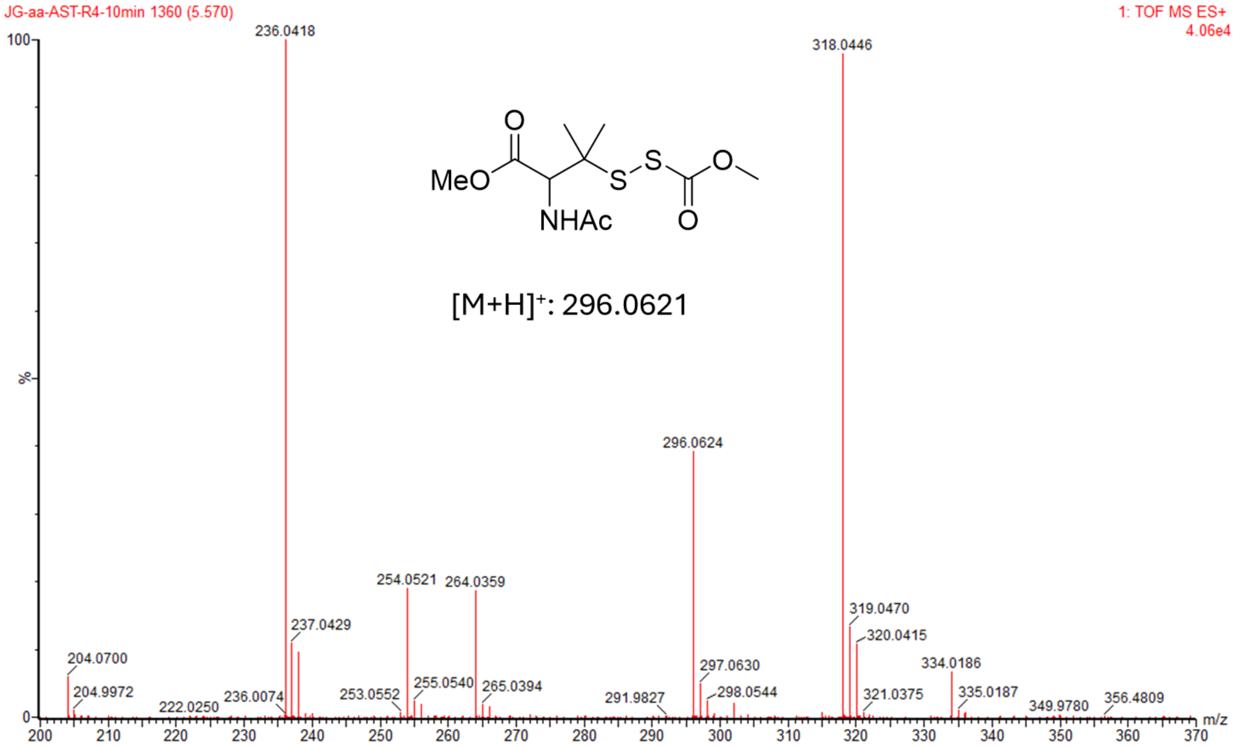

Figure S2. HRMS of the peak eluting at 5.6 min corresponding to **AST-2**.

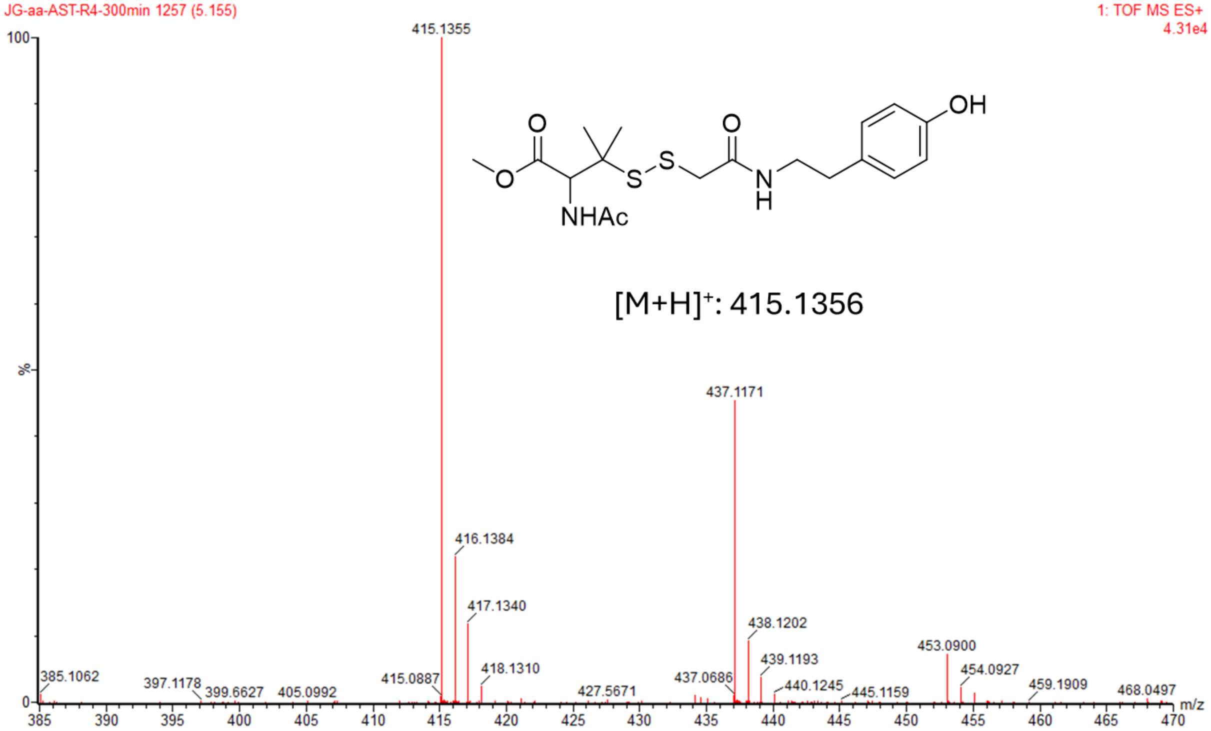

Figure S3. HRMS of the peak eluting at 5.2 min corresponding to **RSS-HPE-AM**.

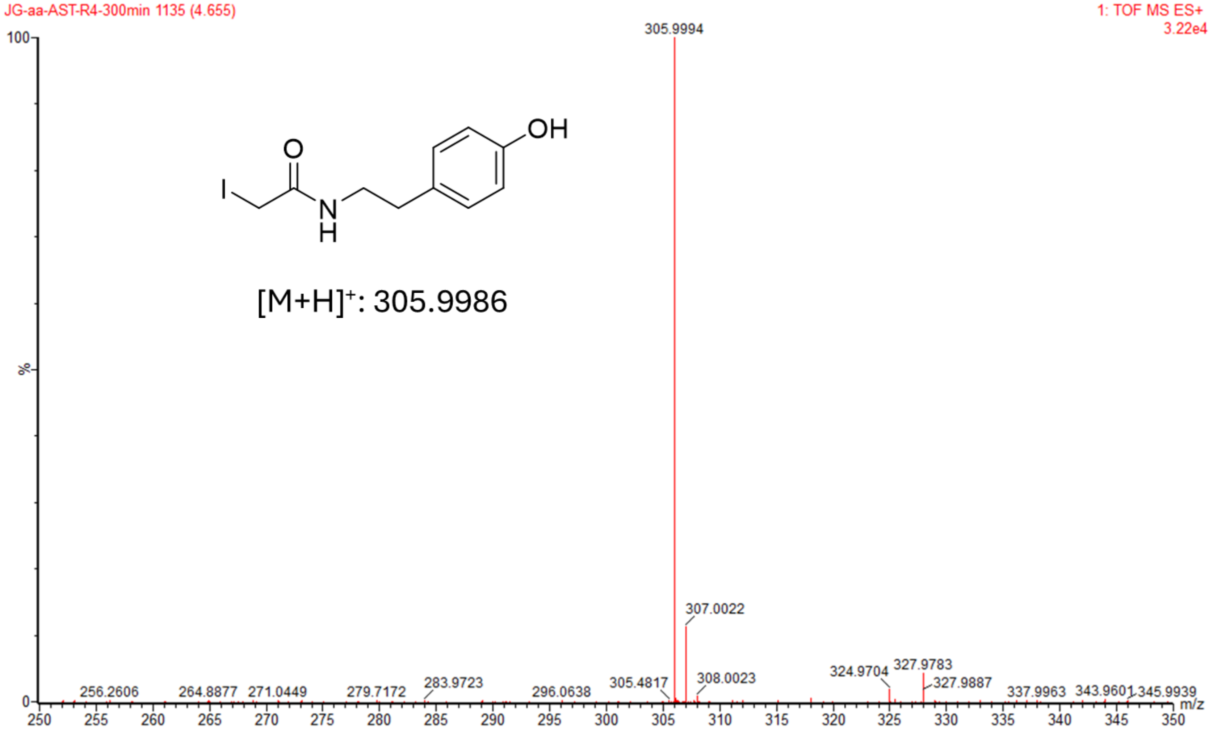

Figure S4. HRMS of the peak eluting at 4.7 min corresponding to **HPE-IAM**.

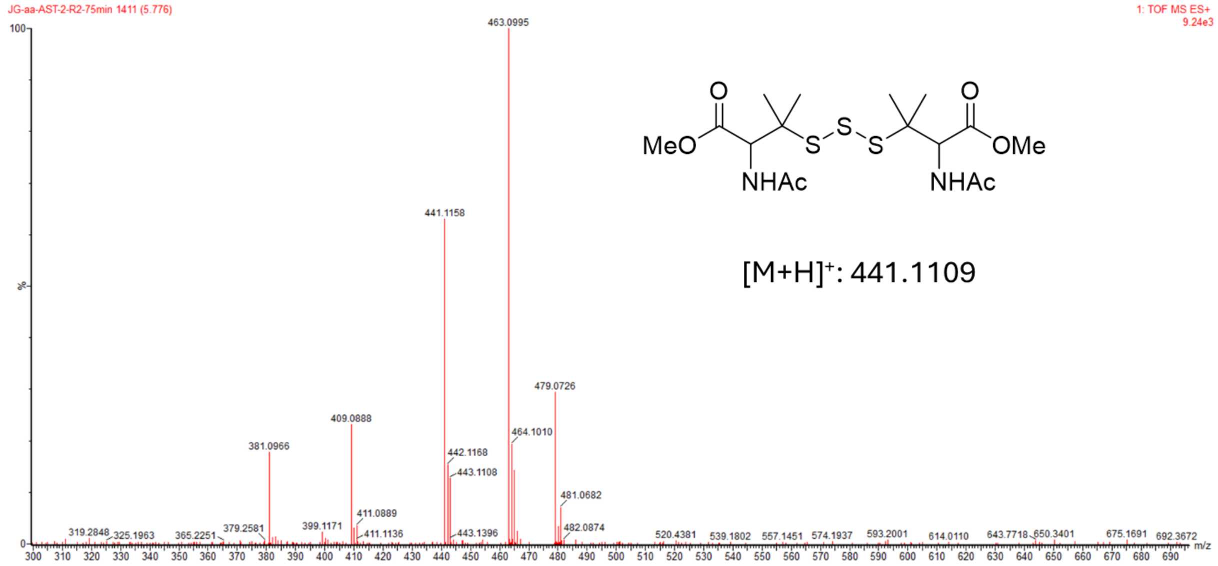

Figure S5. HRMS of the peak eluting at 5.7 min corresponding to dialkyltrisulfide.

Extracted Ion Chromatograms of APT-1 Hydrolysis Monitored by UPLC-MS

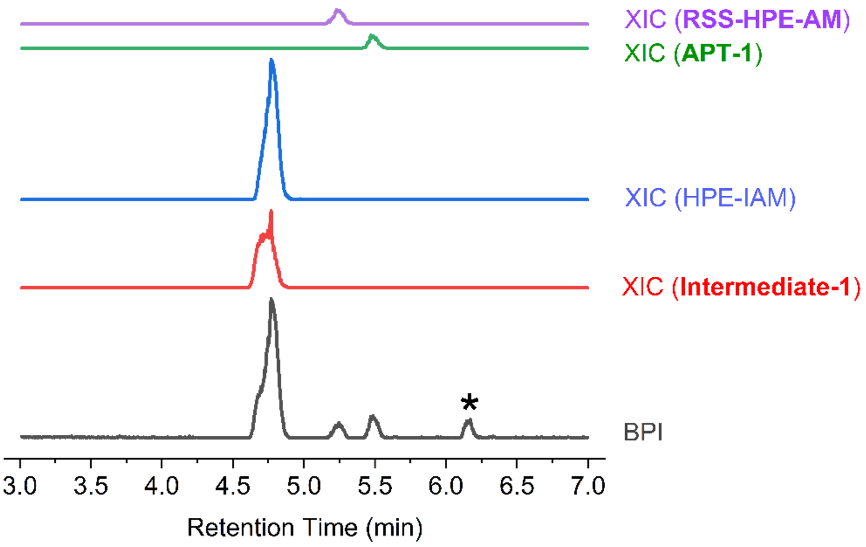

Figure S6. Base peak intensity (BPI) chromatogram of **APT-1** (25 µM) incubated with porcine liver esterase (PLE, 1 U/mL) and HPE-IAM (250 µM) at 37 °C for 60 min (bottom trace). Extracted ion chromatograms (XICs), derived from the same BPI using exact-mass selection, are stacked above for **Intermediate-1** ($[M+H]^+$, *m/z* 339.1043), HPE-IAM ($[M+H]^+$, *m/z* 305.9985), **APT-1** ($[M+H]^+$, *m/z* 381.1149), and **RSS-HPE-AM** ($[M+H]^+$, *m/z* 415.1356). Selective ion extraction demonstrates that the **Intermediate-1** signal is resolved from HPE-IAM.

HRMS Data for Compounds Formed during RSSH Generation from APT-1

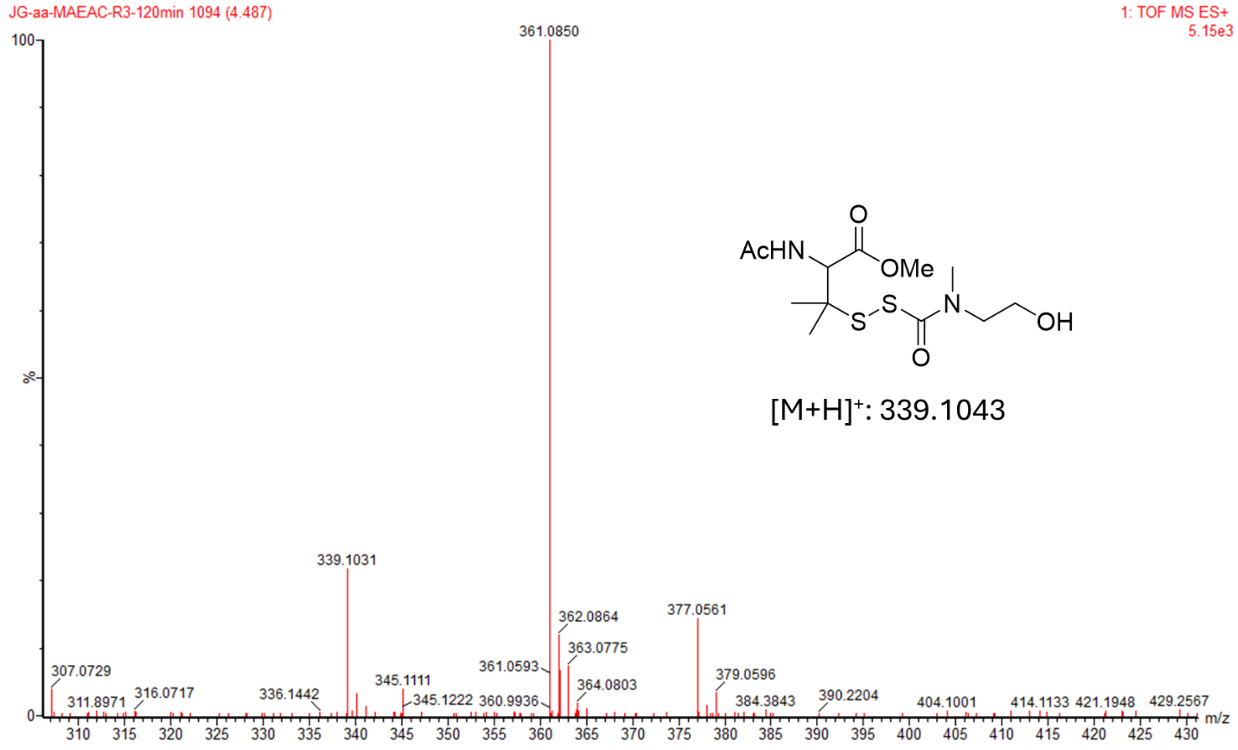

Figure S7. HRMS of the peak eluting at 4.7 min corresponding to **Intermediate-1**.

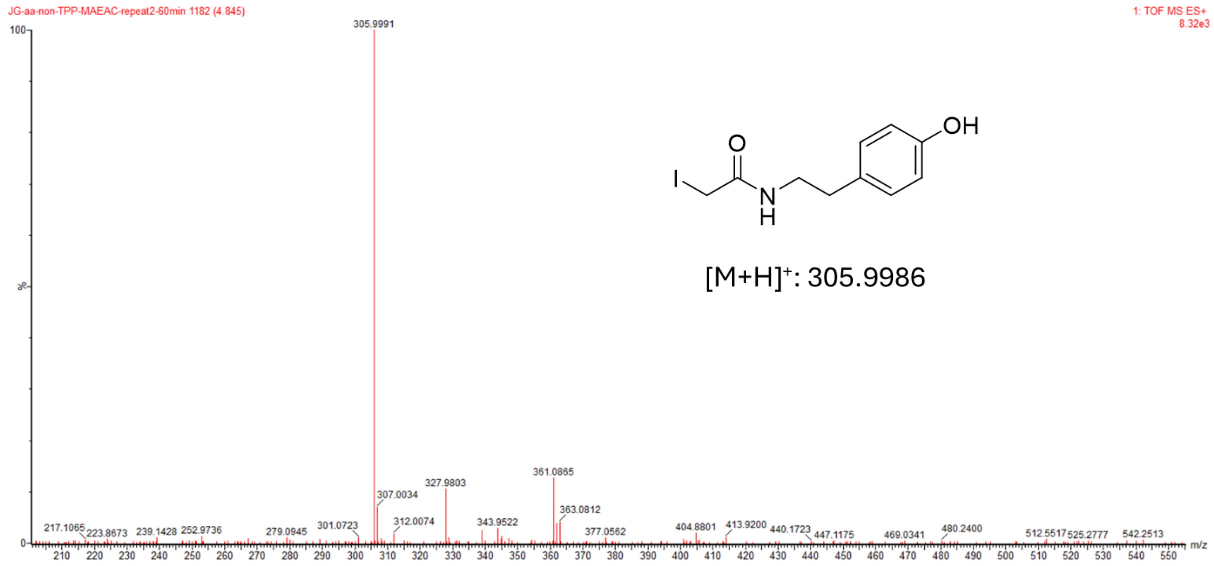

Figure S8. HRMS of the peak eluting at 4.7 min corresponding to **HPE-IAM**.

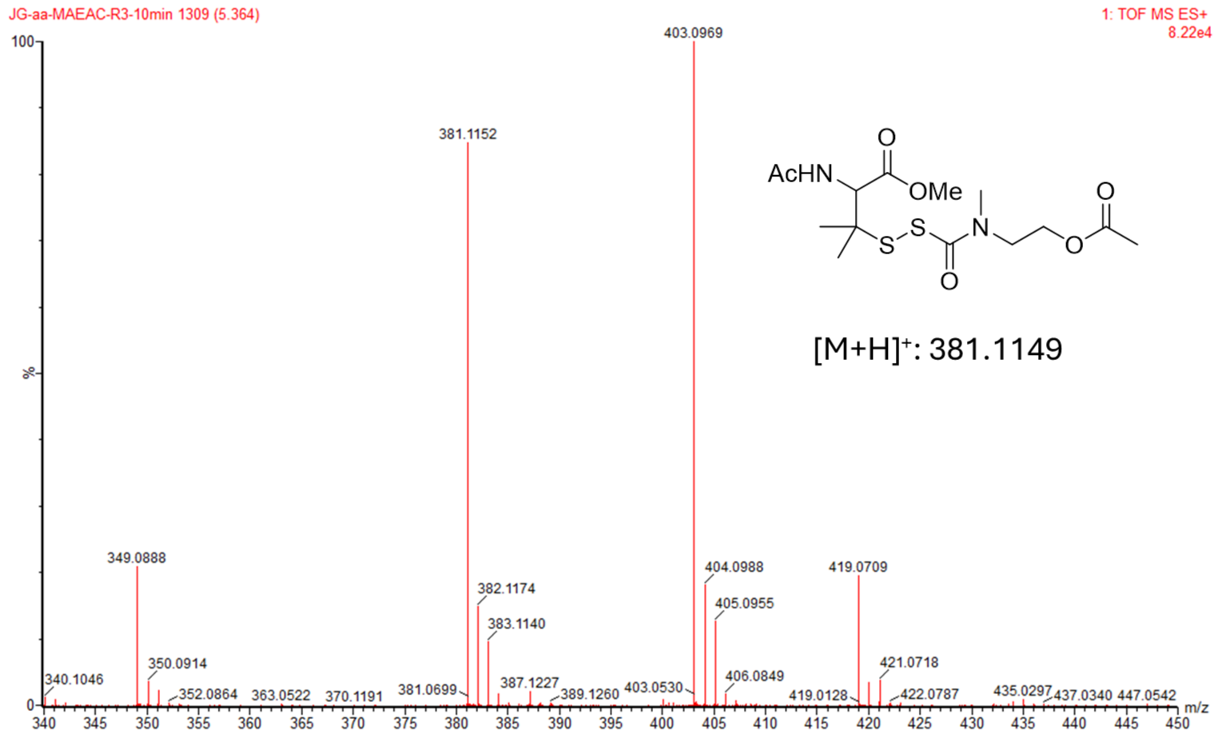

Figure S9. HRMS of the peak eluting at 5.4 min corresponding to **APT-1**.

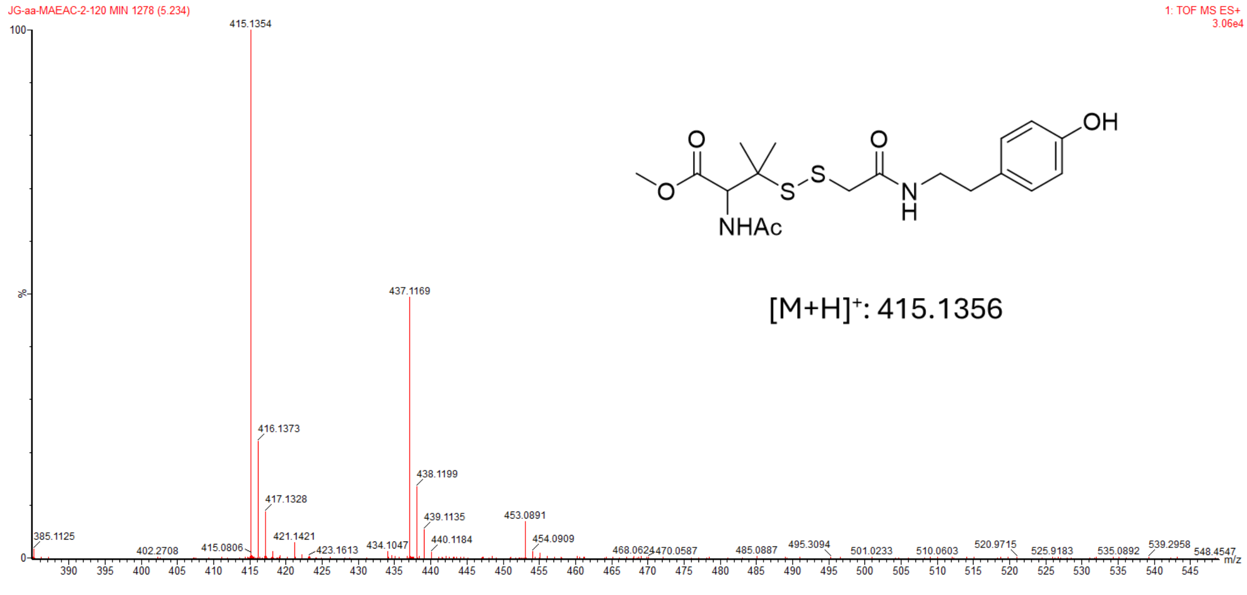

Figure S10. HRMS of the peak eluting at 5.2 min corresponding to **RSS-HPE-AM**.

Esterase-mediated hydrolysis of AST-2-TPP monitored by UPLC-MS

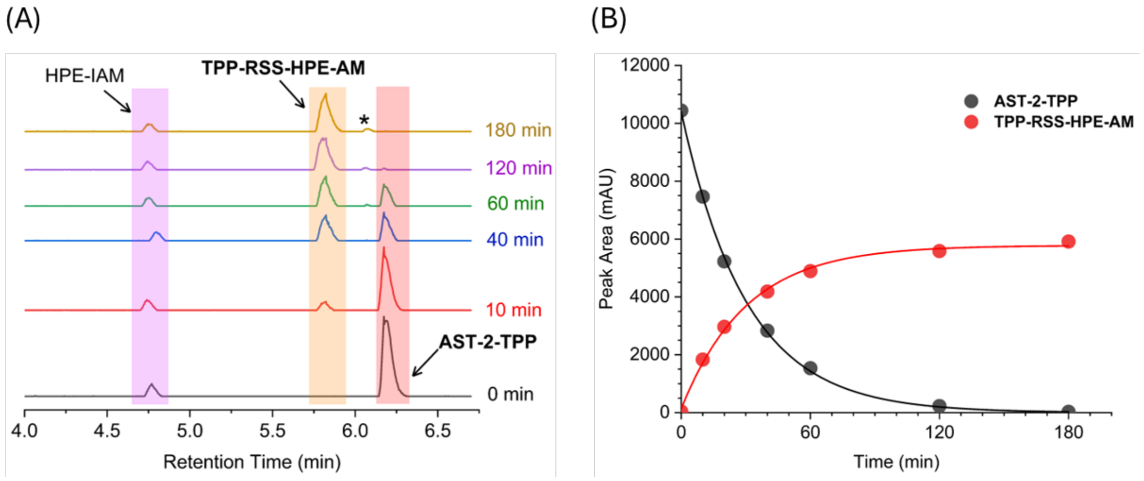

Figure S11. (A) Representative UPLC-MS traces showing esterase-mediated hydrolysis of **AST-2-TPP** (25 μM) in the presence of HPE-IAM (250 μM) in ammonium bicarbonate buffer (pH 7.4, 100 mM) containing the metal chelator DTPA (100 μM) at 37 °C. An aliquot of the reaction mixture was withdrawn at the specified time and quenched with concentrated formic acid and analyzed by UPLC-MS. (B) Representative kinetics of **AST-2-TPP** decomposition and **TPP-RSS-HPE-AM** formation. The curves are the calculated best fit to a single-exponential function. Asterisk represents minor dialkyltrisulfide formation. First-order kinetic analysis of AST-2-TPP consumption (k = 0.0335 ± 0.0010 min^{-1}, $t_{1/2}$ = 20.7 ± 0.6 min, n=3) and RSS-HPE-AM formation (k = 0.03467 ± 0.0028 min^{-1}, $t_{1/2}$ = 20.1 ± 1.6 min. All experiments were performed in triplicate (n = 3), and numerical data are reported as mean ± standard deviation (SD).

HRMS Data for Compounds Formed during RSSH Generation from AST-2-TPP

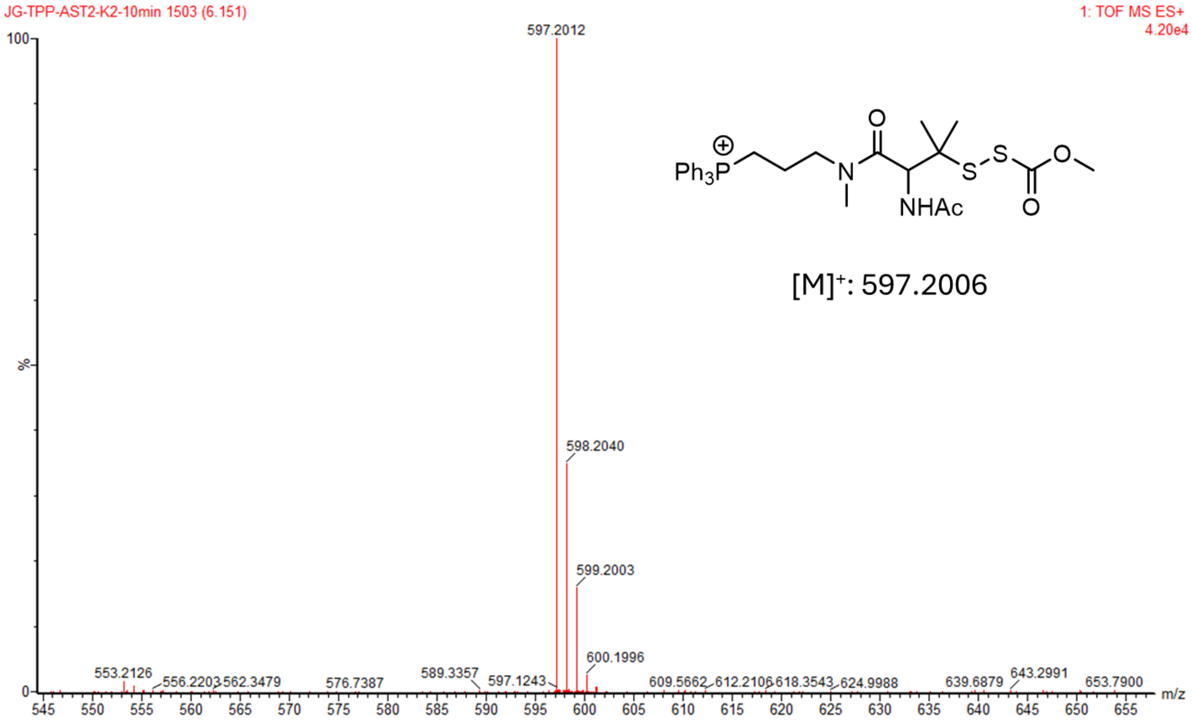

Figure S12. HRMS of the peak eluting at 6.2 min corresponding to **AST-2-TPP**.

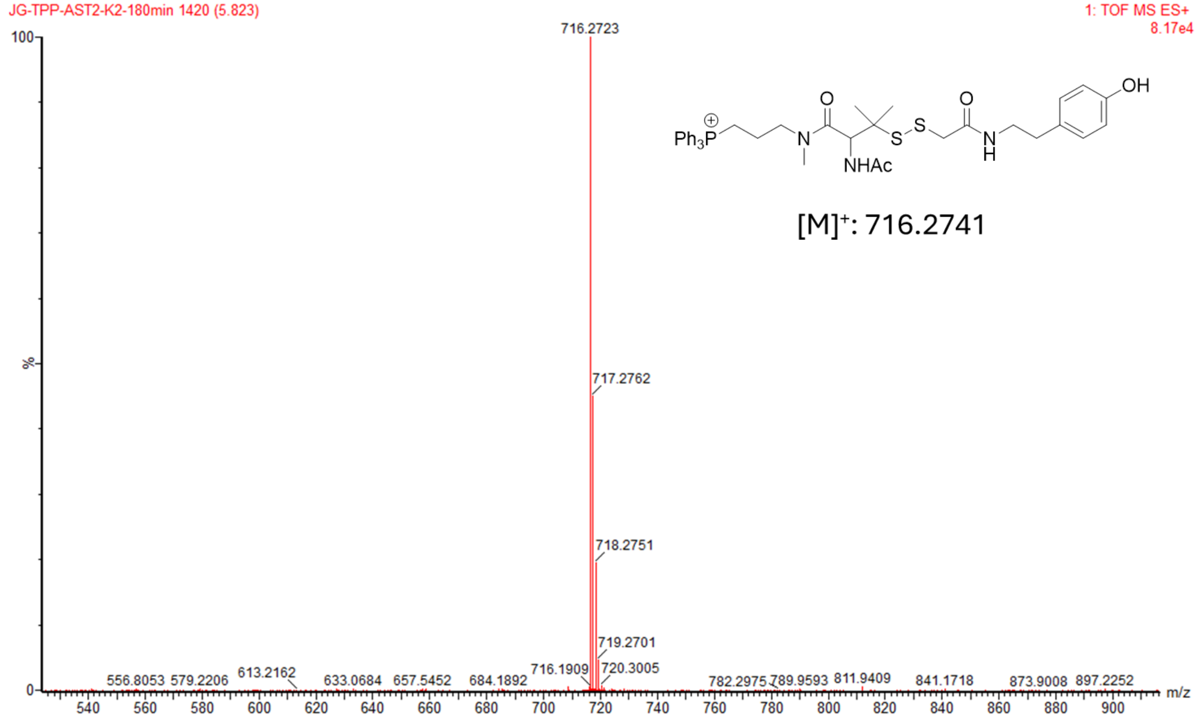

Figure S13. HRMS of the peak eluting at 5.8 min corresponding to **TPP-RSS-HPE-AM**.

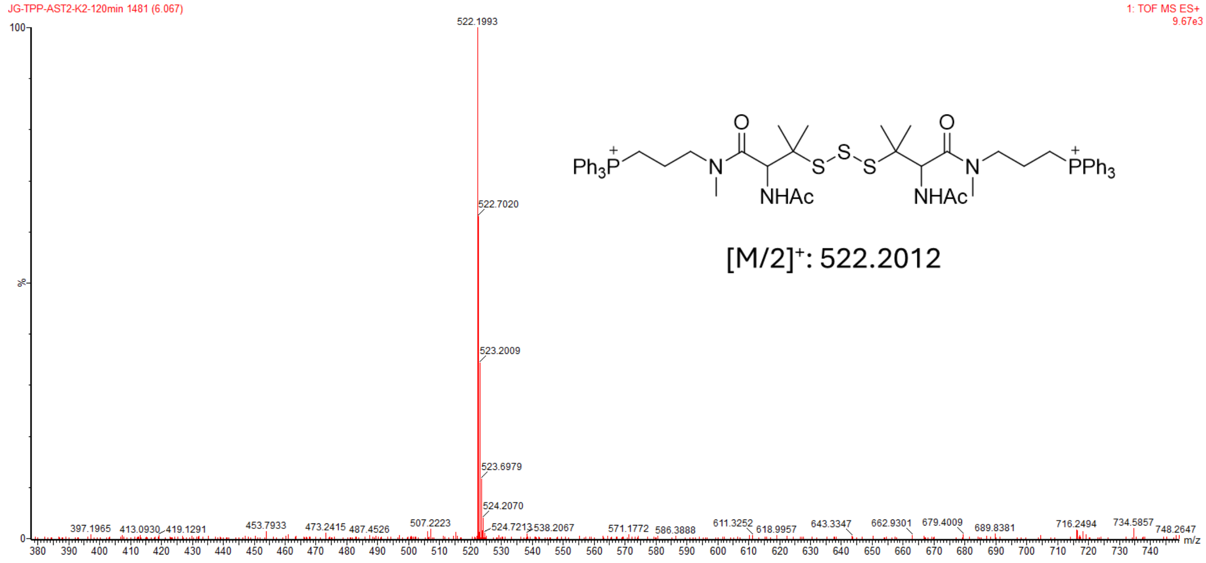

Figure S14. HRMS of the peak eluting at 6.1 min corresponding to dialkyltrisulfide.

Esterase-mediated hydrolysis of APT-1-TPP monitored by UPLC-MS

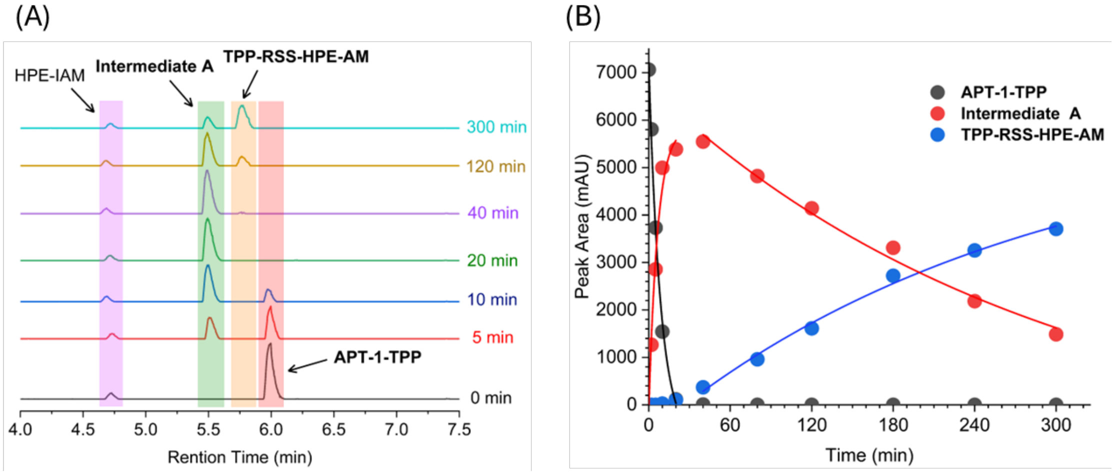

Figure S15. (A) Representative UPLC-MS traces showing esterase-mediated hydrolysis of **APT-1-TPP** (25 μM) in the presence of HPE-IAM (250 μM) in ammonium bicarbonate buffer (pH 7.4, 100 mM) containing the metal chelator DTPA (100 μM) at 37 °C. An aliquot of the reaction mixture was withdrawn at the specified time and quenched with concentrated formic acid and analyzed by UPLC-MS. (B) Representative kinetics of **APT-1-TPP** decomposition, alcohol intermediate formation and decomposition, and **TPP-RSS-HPE-AM** formation. The curves are the calculated best fit to a single-exponential function. First order rate constant of deacetylation step k_1 = 0.1338 ± 0.0049 min^{-1} with $t_{1/2}$ = 5.2 ± 0.2 min; First order rate constant of RSS-HPE-AM formation k_2 = 0.0049 ± 0.0002 min^{-1} with $t_{1/2}$ = 142.5 ± 7.0 min. All experiments were performed in triplicate (n = 3), and numerical data are reported as mean ± standard deviation (SD).

HRMS Data for Compounds Formed during RSSH Generation from APT-1-TPP

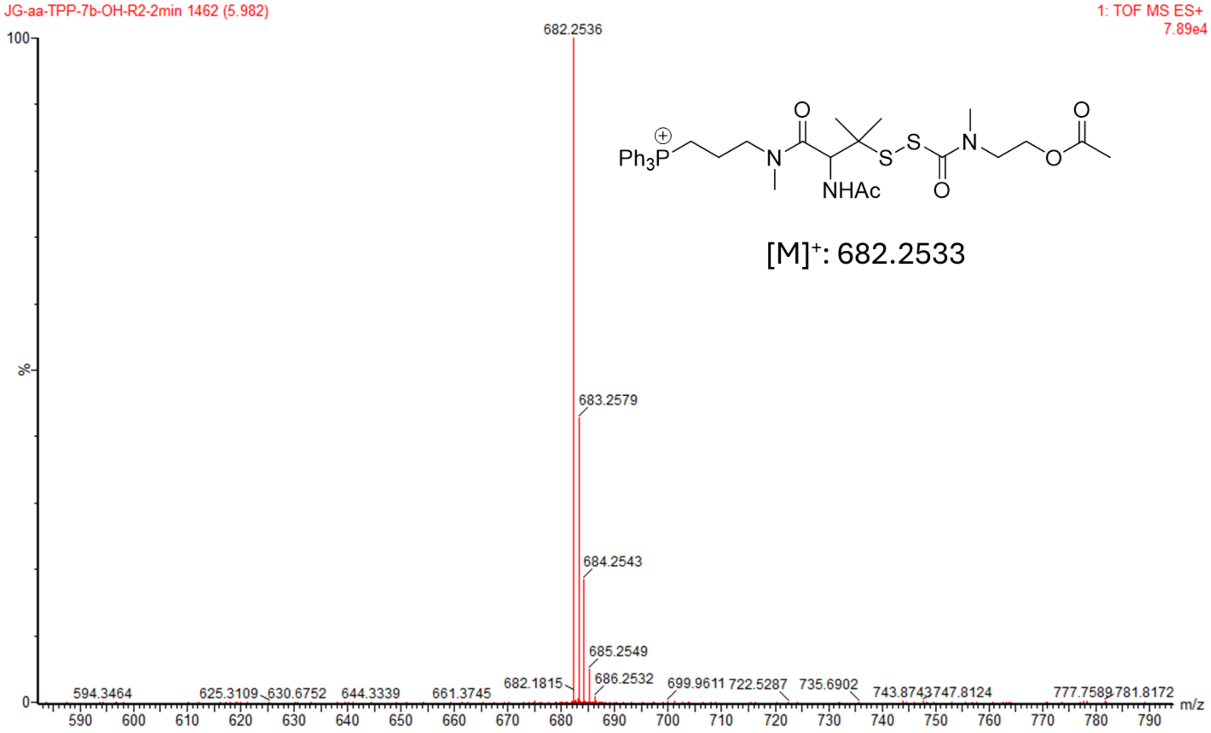

Figure S16. HRMS of the peak eluting at 6.0 min corresponding to **APT-1-TPP**.

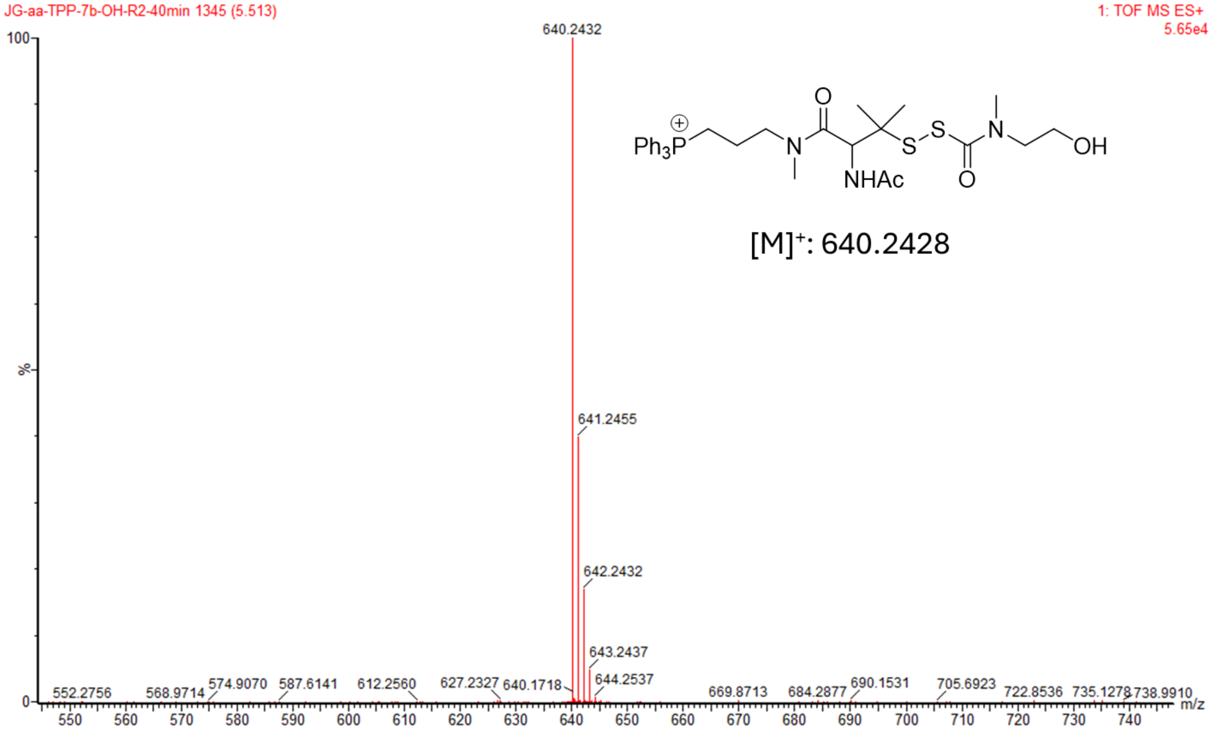

Figure S17. HRMS of the peak eluting at 5.5 min corresponding to **Intermediate A**.

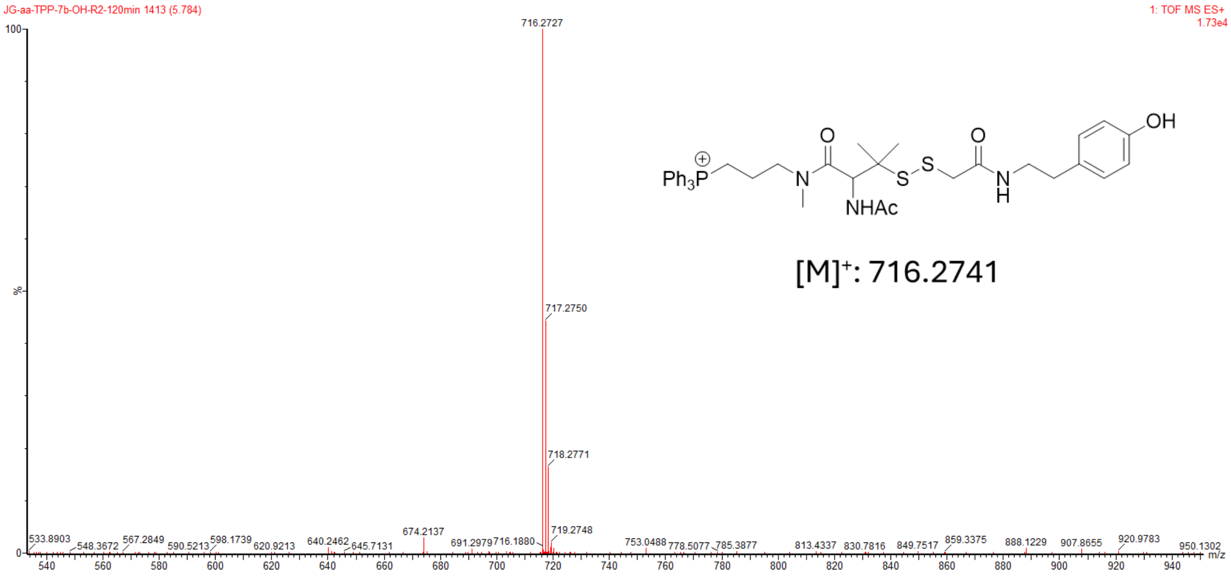

Figure S18. HRMS of the peak eluting at 5.8 min corresponding to **TPP-RSS-HPE-AM**.

C. Quantification of RSSH Release by HPLC Analysis

The RSSH precursor (100 µM) was incubated with HPE-IAM (2 mM) and porcine liver esterase (PLE, 1 U/mL) in ammonium bicarbonate buffer (100 mM, pH 7.4) containing diethylenetriaminepentaacetic acid (DTPA, 100 µM) at 37 °C. At designated time points, 200 µL aliquots were withdrawn and immediately quenched by mixing with 200 µL of ice-cold 0.1% formic acid. Samples were analyzed by HPLC using the same method and conditions as described above. Quantification of the trapped product (RSS-HPE-AM) was carried out using a calibration curve generated in-house with authentic standards. All experiments were performed in triplicate (n = 3), and data are reported as mean ± standard deviation (SD).

Stability of RSSH Precursors with Glutathione by HPLC

Stock solutions of RSSH precursor (10 mM in DMSO) and GSH (20 mM in PBS, pH 7.4) were prepared immediately prior to the assay. The RSSH precursor (100 µM) was incubated with GSH (500 µM) in phosphate-buffered saline (PBS, pH 7.4) at 37 °C. At designated time points, 200 µL aliquots were withdrawn and immediately quenched by mixing with an equal volume of ice-cold 0.1% formic acid. Samples were analyzed by Agilent HPLC with UV detection using a C18 reverse-phase column (Phenomenex Luna C18, 4.6 × 150 mm, 5 µm) at 25 °C with a flow rate of 1.0 mL/min and an injection volume of 20 µL. The gradient elution (all solvents v/v, containing 0.1% trifluoroacetic acid) was as follows: 0–10 min, linear gradient from 90% water / 10% acetonitrile to 50% water / 50% acetonitrile; 10–18 min, to 10% water / 90% acetonitrile; 18–20 min, held at 10% water / 90% acetonitrile; 20–25 min, return to 90% water / 10% acetonitrile. Absorbance was monitored at 275 nm. The percentage of precursor remaining at each time point was calculated by comparing the absorbance at 275 nm to the initial value. All experiments were performed in triplicate (n = 3), and data are reported as mean ± standard deviation (SD).

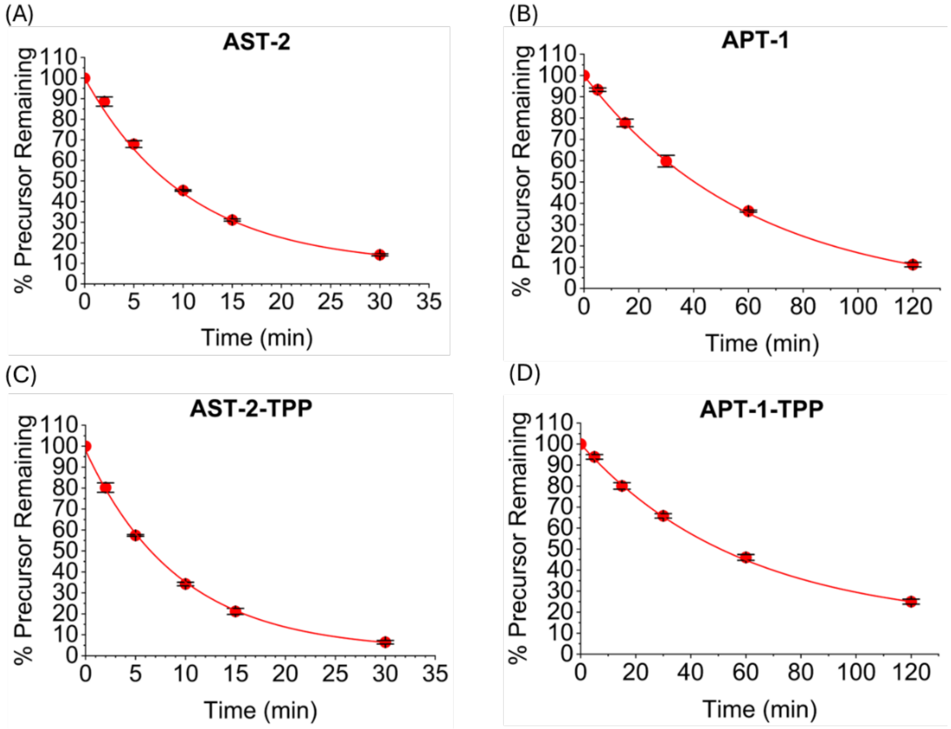

Figure S19. Stability profiles of (A) **AST-2**, (B) **APT-1**, (C) **AST-2-TPP**, and (D) **APT-1-TPP** in PBS, pH 7.4 at 37 °C in the presence of GSH. Decomposition rate constant for **AST-2**, **APT-1**, **AST-2-TPP**, and **APT-1-TPP** are 0.08081 ± 0.00105, 0.01749 ± 0.00052, 0.10937 ± 0.00183, and 0.01236 ± 0.00066, respectively; Decomposition half-lives for **AST-2**, **APT-1**, **AST-2-TPP**, and **APT-1-TPP** are 8.6 ± 0.1 min, 39.7 ± 1.2 min, 6.3 ± 0.1 min, and 56.2 ± 4.0 min, respectively. Data are mean ± standard deviation (SD) (n = 3)

Carbonyl Sulfide Generation from RSSH Precursors with Glutathione by MIMS

COS measurement was performed following a previously reported method.[6] Briefly, MIMS analysis was conducted using a Hiden HPR-40 system equipped with a 20 mL sample cell and a gas-permeable membrane selective for detecting dissolved gases in aqueous solutions. The sample cell was filled with 20 mL of PBS (100 mM, pH 7.4) containing DTPA (100 µM) and purged with argon for at least 30 min prior to analysis. A stock solution of GSH (100 mM) was prepared in DI H_2O, and RSSH precursor stock solutions (20 mM) were prepared in DMSO, purged with nitrogen for 10 min, and used shortly after preparation. Using a gas-tight syringe, 100 µL of the GSH stock solution was injected into the sample cell and allowed to equilibrate for 10 min, followed by the addition of 100 µL of the RSSH precursor. The masses of interest were monitored continuously in positive ion mode. Compound **7**, synthesized as previously reported,[6] was employed as a COS donor.

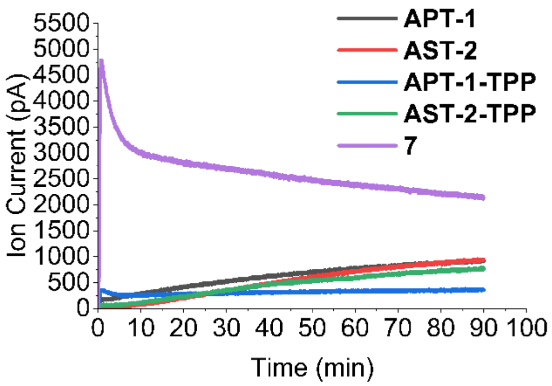

Figure S20. COS MIMS signals at *m/z* 60 observed following the reaction of **AST-2**, **APT-1**, **AST-2-TPP**, **APT-1-TPP,** and compound **7** (100 µM) with GSH (500 µM, 5 equiv.) in PBS (pH 7.4, 10 mM) with DTPA (100 µM) at 37 °C.

Intracellular RSSH Analysis by LC-MS

H9c2 cells were cultured in 150-mm dishes to 80–90% confluency and treated for 2 h with either control medium (FBS-free DMEM, 0.01% DMSO) or 200 µM RSSH donors (FBS-free DMEM with **APT-1** or **APT-1-TPP**). Cells were washed two times with warm PBS, collected by scraping, and centrifuged at 1,000 × g for 10 min at 4 °C (Sorvall ST 8R, ThermoFisher). Pellets were resuspended in 0.6 mL sucrose buffer; ¼ was reserved for BCA assay and ¾ used for fractionation. Cells were lysed on ice by 20 passages through a 26-G needle and centrifuged at 1,500 × g for 10 min at 4 °C (Z 216 MK, HERMLE). Pellets (nuclear/debris fraction) were collected, and supernatants were centrifuged at 12,000 × g for 10 min at 4 °C. Pellets (mitochondrial fraction) and supernatants (cytoplasmic fraction) were collected. Each of these fractions were resuspended in methanol containing 5 mM HPE-IAM, sonicated (3 × 10 s on/10 s off, Branson Sonifier SFX150), incubated at 37 °C for 30 min, and centrifuged at 16,000 × g for 10 min at 4 °C. Supernatants were analyzed by MRM LC-MS/MS (Ultivo Triple Quadrupole, Agilent) using a Poroshell 120 EC-C18 column (4.6 × 100 mm, 2.7 µm; Agilent) at 30 °C, 0.6 mL/min, with the following gradient (solvent A: water + 0.1% TFA; solvent B: acetonitrile + 0.1% TFA): 0–1.5 min, 97% A; 1.5–7.5 min, linear to 5% A; 7.5–8.0 min, 5% A; 8.0–8.01 min, return to 97% A; 8.01–13.0 min, 97% A. Injection volume: 10 µL. Experiments were performed in triplicate (n = 3 biological replicates). Data are shown as mean ± SEM.

MRM Parameters for LC-MS/MS Source Parameters: Gas Flow: 13.0 L/min; Nebulizer: 35.0 psi; Sheath Gas Flow: 11.0 L/min; Capillary Voltage: Positive Setpoint 4000 V, Negative Setpoint 4000 V; Nozzle Voltage: Positive Setpoint 1500 V, Negative Setpoint 1500 V; Gas Temperature: 300 °C; Sheath Gas Temperature: 250 °C

Table S1. MRM parameters for LC-MS/MS

Analyte	Polarity	Precursor ion (m/z)	Product ion (m/z)	Collision energy (V)
Bis-S-HPE-AM	+	389.1	121.0	30
Bis-SS-HPE-AM	+	421.1	121.0	23
Cys-SS-HPE-AM	+	331.1	121.0	29
GSS-HPE-AM	+	517.1	388.2	18
Pen-SS-HPE-AM	+	415.1	354.8	22
Cys-S-HPE-AM	+	299.1	121.0	29
GS-HPE-AM	+	485.2	356.3	18

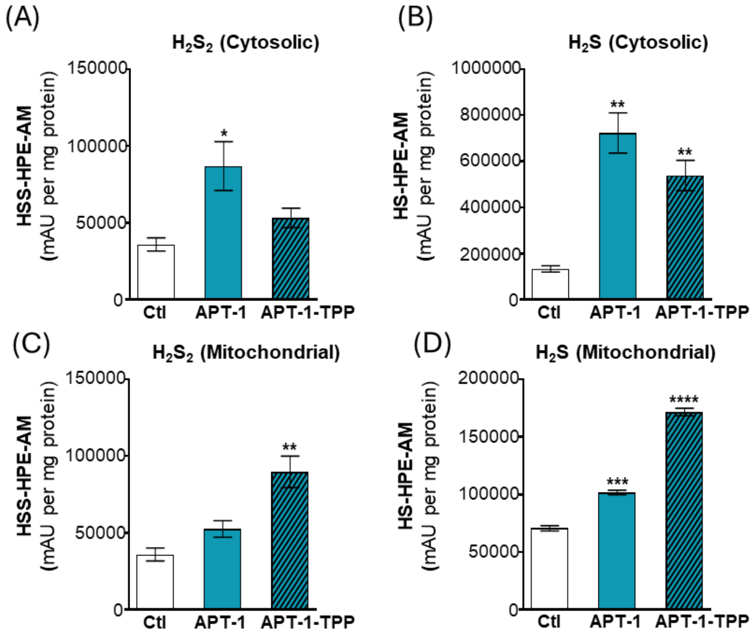

Figure S21. Measurement of H_2S_2, and H_2S in cytoplasm (A, B) and mitochondria (C,D) of H9c2 cells treated with **APT-1** (200 μM) **APT-1-TPP** (200 μM), or vehicle (0.01% DMSO in serum-free medium) for 2 h. Data are mean ± SEM (n = 3); *p ≤ 0.05, **p ≤ 0.01.

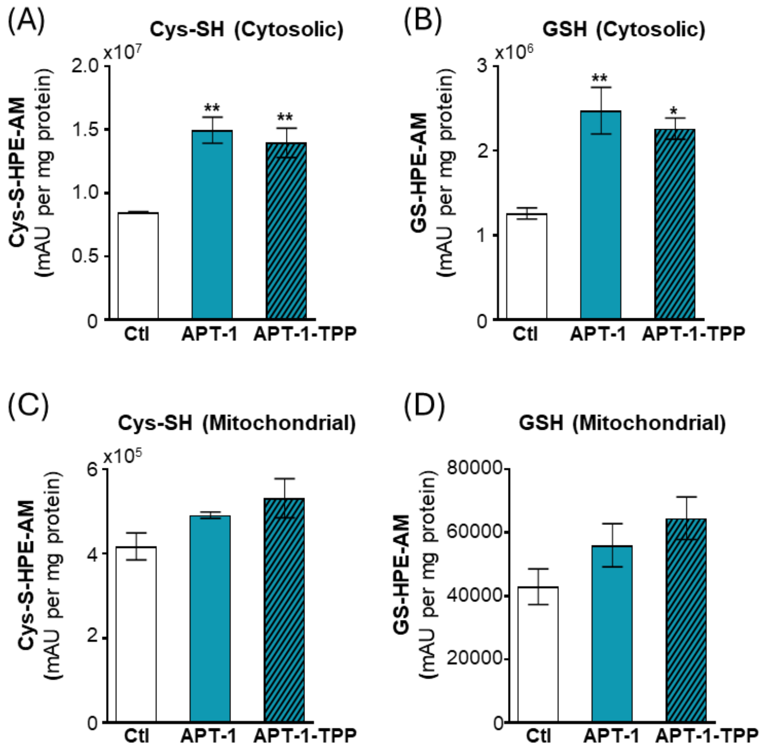

Figure S22. Measurement of Cys-SH and GSH in cytoplasm (A, B) and mitochondria (C, D) of H9c2 cells treated with **APT-1** (200 μM) **APT-1-TPP** (200 μM), or vehicle (0.01% DMSO in serum-free medium) for 2 h. Data are mean ± SEM (n = 3); *p ≤ 0.05, **p ≤ 0.01.

Cell Viability Study

Cytotoxicity Assessment of Hydropersulfide Precursors in H9c2 and HepG2 cells

Cell viability was measured using the Cell Counting Kit-8 (CCK-8, Dojindo).[7] H9c2 cardiomyoblast and HepG2 cells were seeded at a density of 8 × 10^3 cells/well in a 96-well plate (flat-bottom, tissue culture-treated). After 24 h of incubation at 37 °C in a humidified incubator with 5% CO_2, the medium was replaced with 200 μL of fresh growth medium (DMEM with 10% FBS) containing the RSSH donors with varying concentrations. All treatments were prepared with a final DMSO concentration of <0.01%. In the vehicle control group, cells were treated with medium (DMEM with 10% FBS) containing 0.01% DMSO only. Cells were incubated with RSSH donors for an additional 24 h. After 24 h, cells were gently washed three times with PBS, pH 7.4. Then, 100 μL of serum-free medium containing 10% v/v CCK-8 solution was added to each well. The plates were then incubated for 1-2 h before measuring absorbance at 450 nm using a plate reader (SPARK Part No. 30124664 Version 1.7, serial No. 1909010319). Relative cell viability (%) was calculated as: % Viability = 100 × [Abs_{450} (treated cells) / Abs_{450} (vehicle control)]. Each treatment condition was performed in 6 technical replicates per biological replicate, and the experiment was independently repeated three times (n = 3 biological replicates). Data are presented as mean ± SEM.

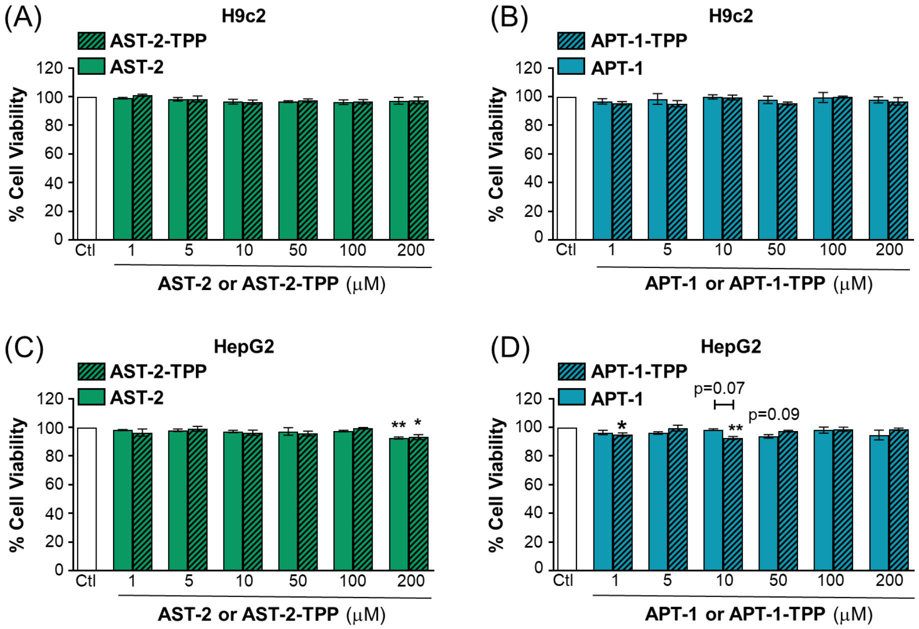

Figure S23. Impact of (A) **AST-2** or **AST-2-TPP** (0-200 μM) and (B) **APT-1** (0-200 μM) or **APT-1-TPP** (0-200 μM) on H9c2 cardiomyocytes. Impact of (C) **AST-2** or **AST-2-TPP** (0-200 μM) and (D) **APT-1** (0-200 μM) or **APT-1-TPP** (0-200 μM) on HepG2 (liver cancer) cells. Results are expressed as mean ± SEM, n≥3; *P<0.05, **P<0.01, ***P<0.001, ****P<0.0001. Ctl = vehicle (medium with 0.01% DMSO) treated cells.

Cytoprotective Effects of RSSH Donors against Doxorubicin Toxicity

Cytoprotective studies were performed as previously described.[8] Briefly, H9c2 cells, HepG2 cells, MDA-MB-468 cells, and MCF-7 cells were seeded at a density of 7 x 10^3 cells/well. After 24 h, the medium was replaced with 190 μL fresh medium (DMEM with 10% FBS) containing varying concentrations of RSSH donors, with DMSO (<0.01%). Additionally, in the vehicle control group, cells were treated with medium (DMEM with 10% FBS) containing 0.01% DMSO only. The cells were then further incubated for an additional 4 h before being treated with DOX (10 μL/well, with final concentration of 5 μM). After a 24 h co-incubation with DOX, each well was carefully washed three times with PBS (pH 7.4) and 100 μL of FBS-free medium containing 10% v/v CCK-8 (Dojindo) was added. The plates were then incubated for 1-2 h before measuring absorbance at 450 nm using a plate reader (SPARK Part No. 30124664 Version 1.7, serial No. 1909010319). The relative % cell viability was calculated as 100 times the ratio of Abs_{450} (RSSH donor + DOX-exposed) to Abs_{450} (vehicle, non-DOX-exposed). Each treatment condition was performed in 6 technical replicates per biological replicate, and the experiment was independently repeated three times (n = 3 biological replicates). Data are presented as mean ± SEM.

Basal Sulfane Sulfur Measurements

Basal sulfane sulfur levels were quantified using the fluorescent probe Sulfobiotics SSP4 (Dojindo, SB10) as previously described.[5] H9c2 cells, HepG2 cells, MDA-MB-468 cells, and MCF-7 cells were seeded in 6-well plates at 1.0 × 10^5 cells per well in 2 mL of DMEM with 10% FBS and incubated at 37 °C for 24 h. When cultures reached ~80% confluency, the medium was replaced with 0.8 mL per well of medium (DMEM with 10% FBS) containing SSP4 (10 μM) and cetyltrimethylammonium bromide (CTAB, 100 μM), and cells were incubated for 30 min at 37 °C. After incubation, cells were washed three times with warm PBS. Fluorescence was recorded on a SpectraMax microplate reader (Molecular Devices) at λ_{ex} = 482 nm and λ_{em} = 515 nm. Following measurement, PBS was removed; cells from the same wells were trypsinized and collected for

counting. Fluorescence signals were normalized by dividing the raw fluorescence units by the corresponding cell number from each well. Data are presented as mean ± SEM.

Mitochondrial Membrane Potential Measurements

Mitochondrial membrane potential was measured using Image-iT™ TMRM Reagent (Cat#I34361, Invitrogen, USA) according to the manufacturer's protocols. For basal mitochondrial membrane potential measurement, H9c2 cells, HepG2 cells, MDA-MB-468 cells, and MCF-7 cells were seeded at a density of 80,000 cells per chamber in a Nunc Lab-Tek Chamber Slide system (ThermoFisher Scientific, Cat#155382PK) and then incubated in growth medium (DMEM with 10% FBS) for 24 h.

For DOX and RSSH related mitochondrial membrane potential measurement, H9c2 cells and HepG2 cells were seeded at a density of 80,000 cells per chamber in a Nunc Lab-Tek Chamber Slide system (ThermoFisher Scientific, Cat#155382PK, one cell line per system, 4 chambers per system, chamber 1 is control, chamber 2 is DOX alone, chamber 3 is **APT-1** + DOX, chamber 4 is **APT-1-TPP** + DOX). Upon seeding each cell line, medium in chamber 1 was replaced with 950 µL vehicle-treated fresh complete medium (DMEM with 10% FBS, containing 0.01% DMSO); medium in chamber 2 was replaced with 950 µL fresh complete medium (DMEM with 10% FBS) cells in chamber 3 and chamber 4 were treated with 950 µL fresh medium (DMEM with 10% FBS) containing RSSH donors (with a final concentration of 25 µM after DOX addition) and incubated for 4 h. After 4 h, 50 µL fresh medium (DMEM containing 10% FBS) was added to chamber 1, 50 µL fresh DMEM medium (containing 10% FBS) and DOX (final concentration of 5 µM) were added to chamber 2, 3 and 4. The cells were incubated for an additional 24 h. After completion of treatment, cells were washed with a warm clear DMEM medium (with 10% FBS) and incubated in culture media containing TMRM (working solution 100 nM) for 30 min at 37 °C. Cells were then washed with a warm clear FBS-free DMEM medium (3 times) before imaging. Images were obtained with Zeiss LSM 800 with AiryScan Confocal (λ_{ex} = 548 nm, λ_{em} = 573 nm). During each experiment, 6 field images were obtained for each experimental group. Images were analyzed using Image J software, and all cells in each image were selected for fluorescence intensity measurement (cell-free space was also measured as blank). H9c2 signal intensity was set to 100% and signal intensity for all the other cell lines was shown as a percentage of that of H9c2. The experiments were independently repeated three times (n = 3 biological replicates). Data are presented as mean ± SEM.

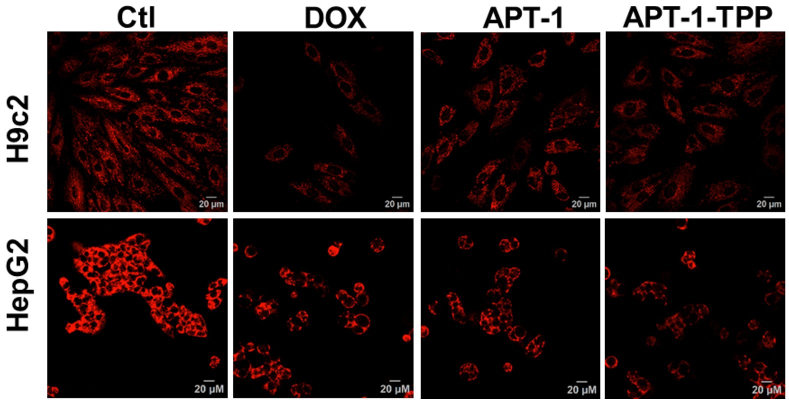

Figure S24. Representative TMRM images for mitochondrial membrane potential (MMP) in H9c2 and HepG2 cells with **APT-1** and **APT-1-TPP** treatment under DOX stress. Ctl = vehicle (medium with 0.01% DMSO) treated cells.

ATP Production Measurements

ATP generation was measured using The CellTiter-Glo® Luminescent ATP Production Assay (Promega Corporation), according to manufacturer instructions [Promega, Catalog #: G9242]. Briefly, H9c2 cells and HepG2 cells were seeded in 96-well white-bottom plate (Alkali Scientific, SKU:TPW9096, TC-treated) at 5000 cells/well. After treatment (Ctrl, 5 μM DOX alone, 4 h pretreatment of 25 μM **APT-1** + 24 h cotreatment with 5 μM DOX, and 4 h pretreatment of 25 μM **APT-1-TPP** + 24 h cotreatment with 5 μM DOX, in clear DMEM medium with 10% FBS), cells were equilibrated at rt for 30 min before CellTiter-Glo® reagent was added to each well. Plates were then placed on an orbital shaker for 2 min and incubated at rt for another 20 min. Chemiluminescence was measured using Tecan plate reader (SPARK Part No. 30124664 Version 1.7, serial No. 1909010319). Cellular ATP concentration was calculated based on the ATP calibration curve and normalized to cell count. The final unit was expressed as ATP concentration in nM per 10^4 cells. Each treatment condition was performed in 6 technical replicates per biological replicate, and the experiment was independently repeated three times (n = 3 biological replicates). Data are presented as mean ± SEM.

References

[1] J. E. Yap, N. Mallo, D. S. Thomas, J. E. Beves, M. H. Stenzel, *Polym. Chem.* **2019**, *10*, 6515-6522.

[2] E. Biavardi, M. Favazza, A. Motta, I. L. Fragalà, C. Massera, L. Prodi, M. Montalti, M. Melegari, G. G. Condorelli, E. Dalcanale, *J. Am. Chem. Soc.* **2009**, *131*, 7447-7455.

[3] D. A. Riccio, P. N. Coneski, S. P. Nichols, A. D. Broadnax, M. H. Schoenfisch, *ACS Appl. Mater. Interfaces* **2012**, *4*, 796-804.

[4] V. S. Khodade, B. M. Pharoah, N. Paolocci, J. P. Toscano, *J. Am. Chem. Soc.* **2020**, *142*, 4309-4316.

[5] V. S. Khodade, Q. Liu, C. Zhang, G. Keceli, N. Paolocci, J. P. Toscano, *J. Am. Chem. Soc.* **2025**, *147*, 7765-7776.

[6] V. S. Khodade, S. C. Aggarwal, B. M. Pharoah, N. Paolocci, J. P. Toscano, *Chem. Sci.* **2021**, *12*, 8252-8259.

[7] M. Ishiyama, Y. Miyazono, K. Sasamoto, Y. Ohkura, K. Ueno, *Talanta* **1997**, *44*, 1299-1305.

[8] B. M. Pharoah, C. Zhang, V. S. Khodade, G. Keceli, C. McGinity, N. Paolocci, J. P. Toscano, *Redox Biol.* **2023**, *60*, 102625.

NMR Spectra:

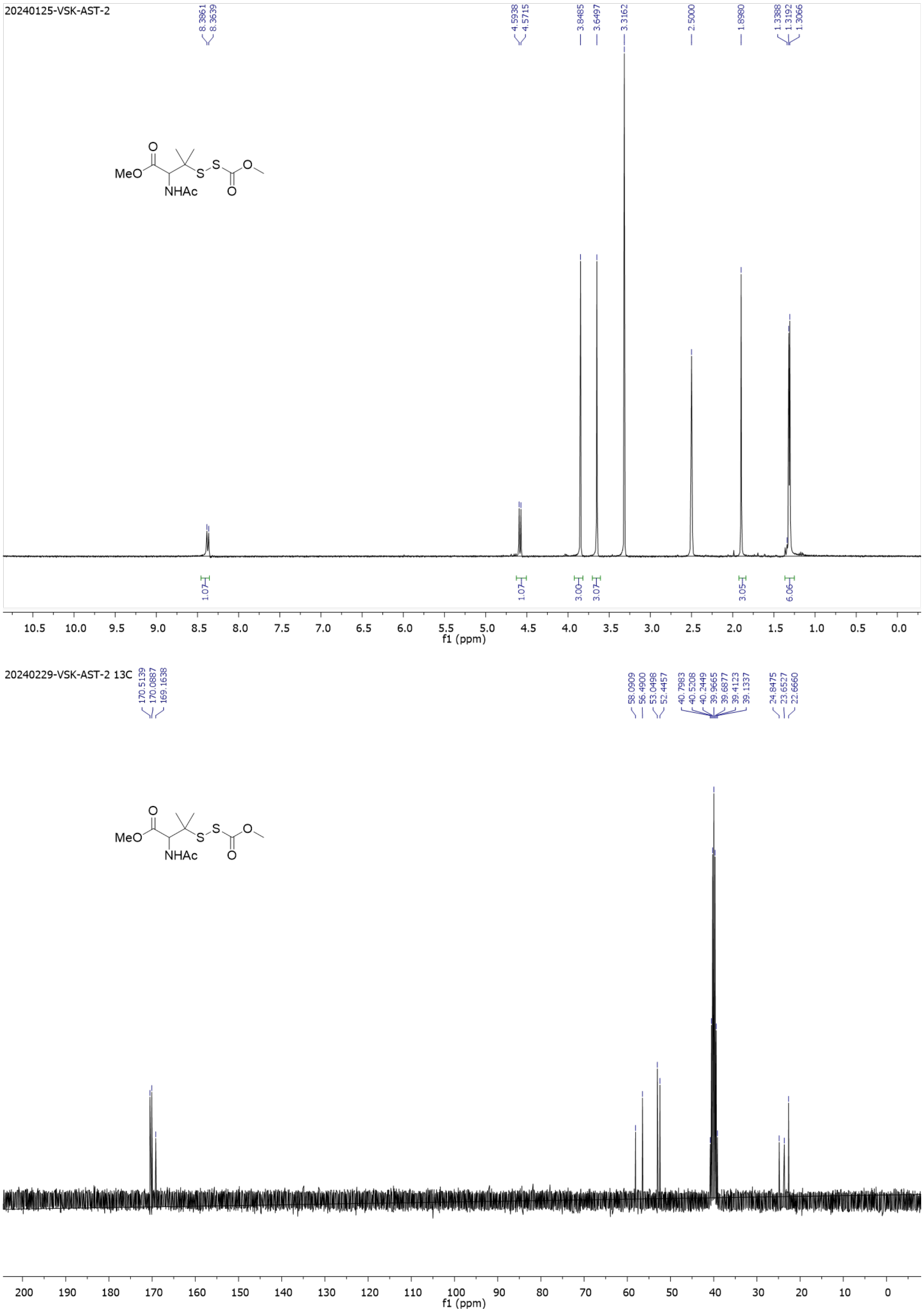

Figure S25. ^{1}H and ^{13}C NMR spectra of **AST-2**.

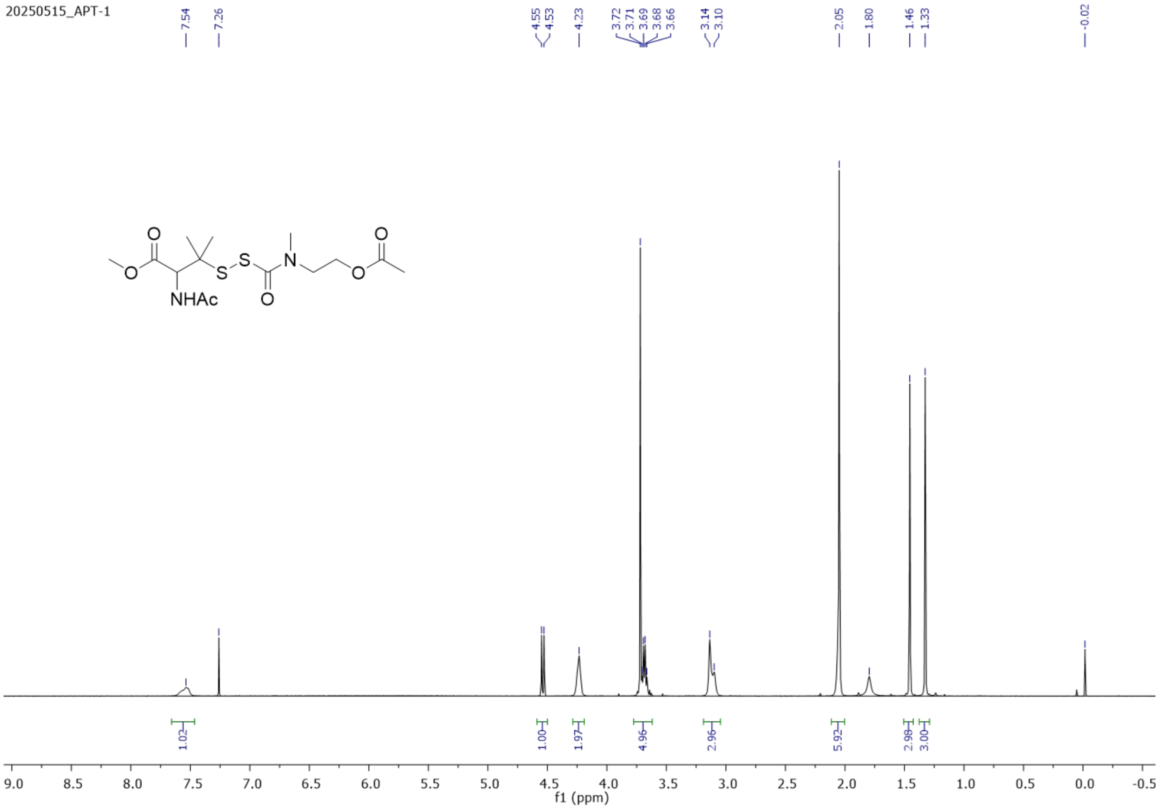

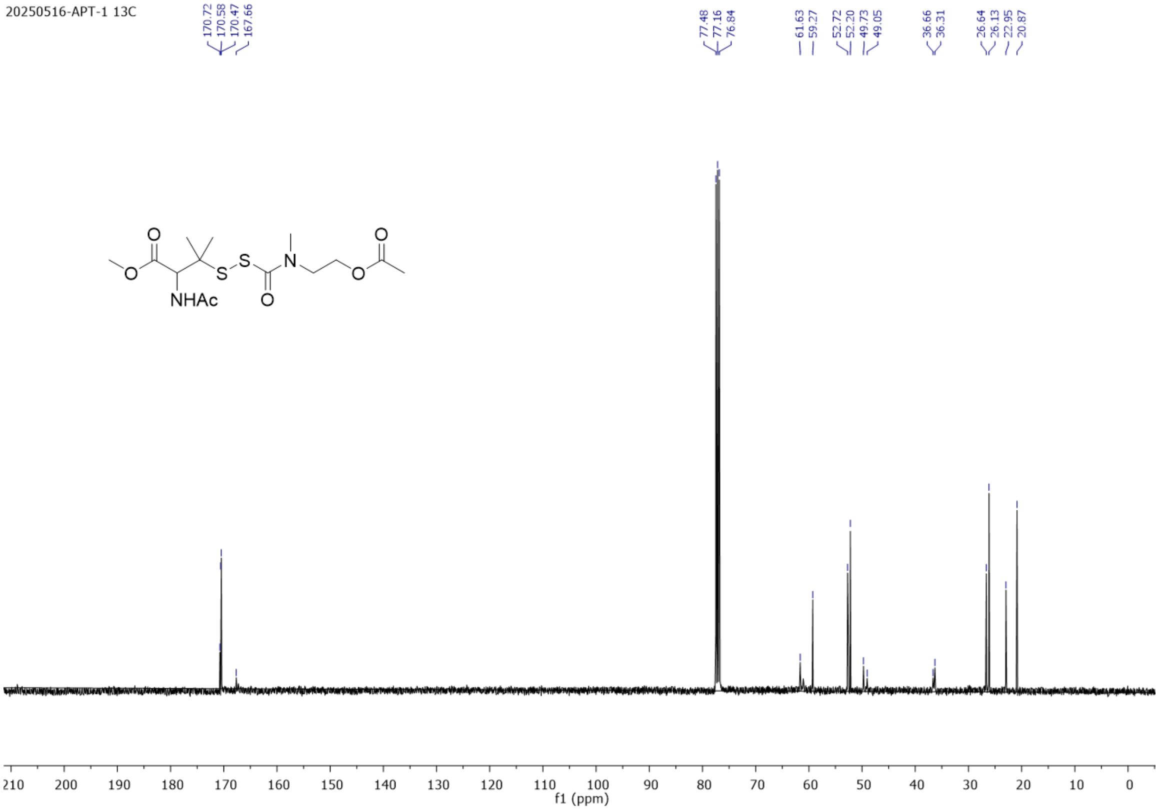

Figure S26. ^{1}H and ^{13}C NMR spectra of **APT-1**.

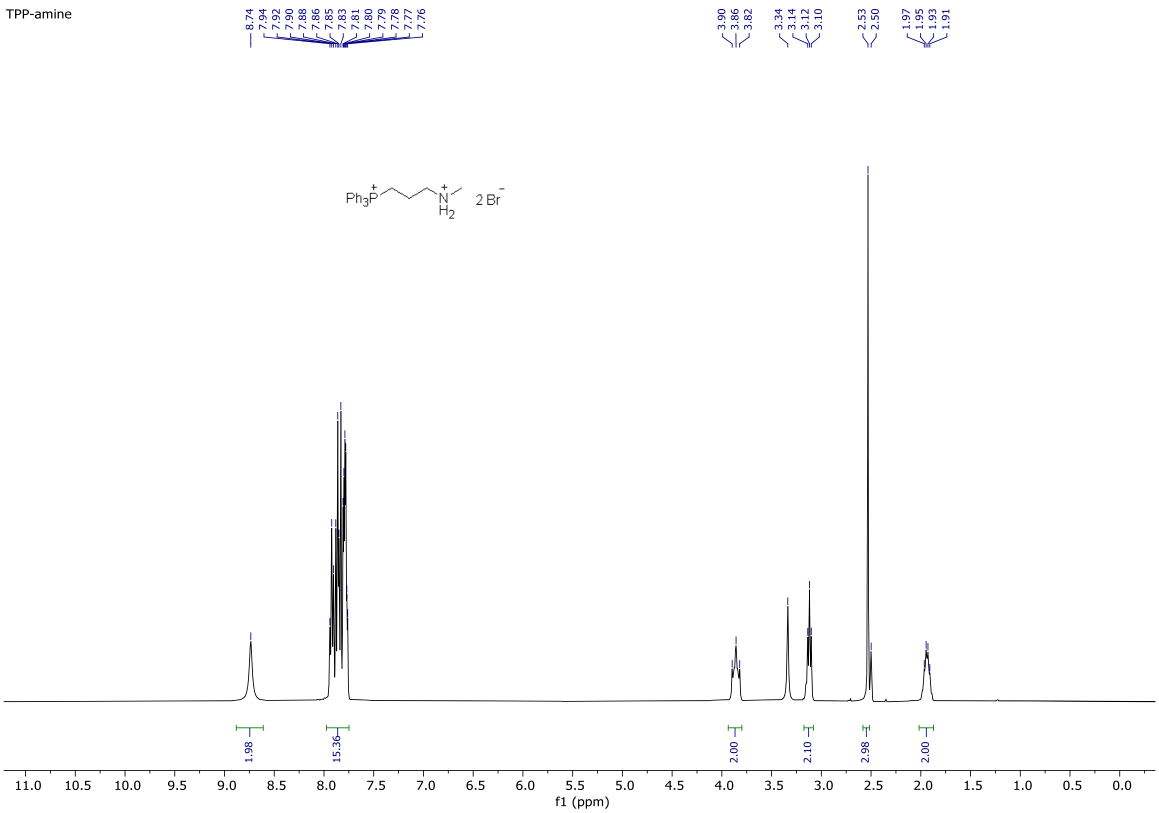

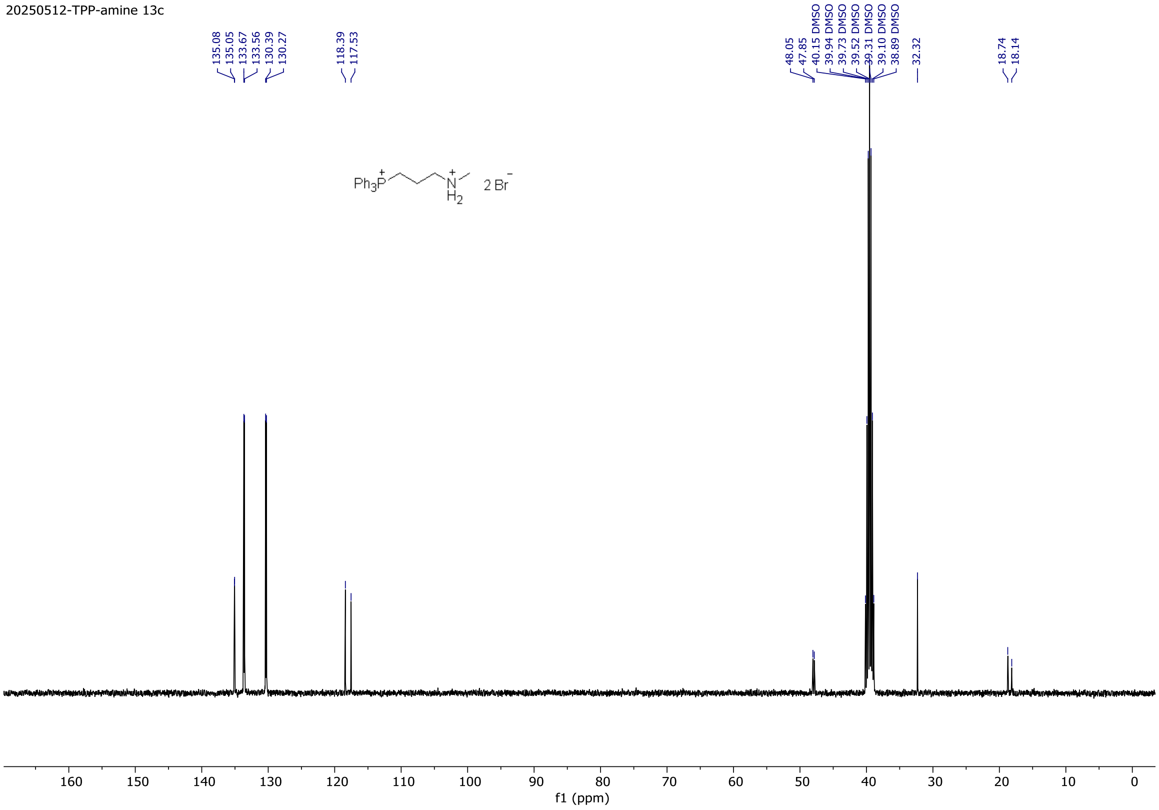

Figure S27. ^1H and ^{13}C NMR spectra of **2**.

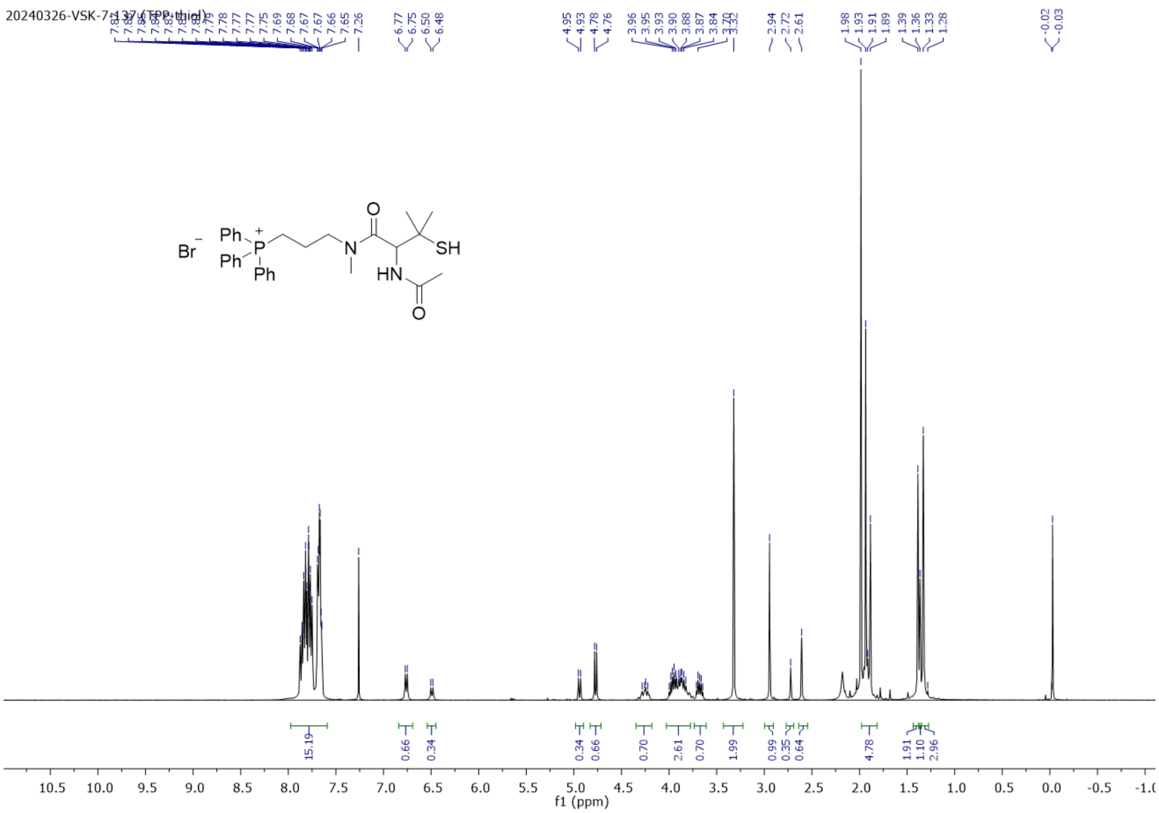

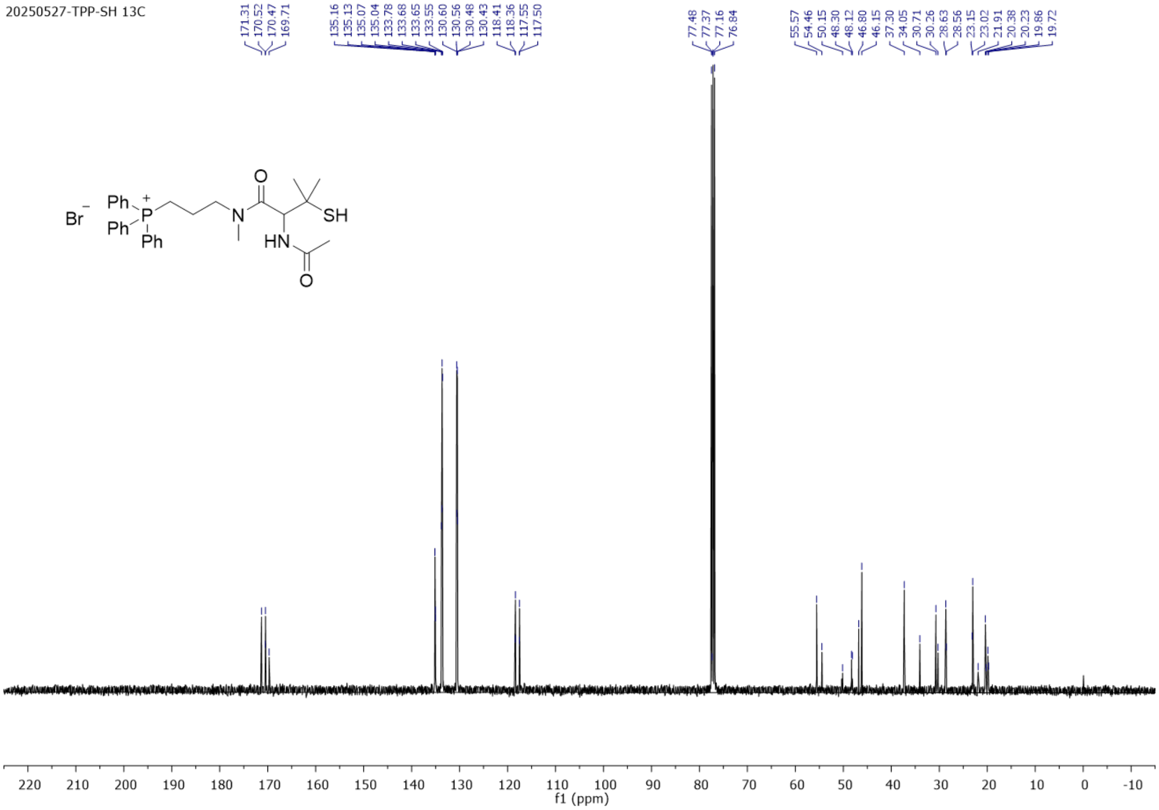

Figure S28. ^{1}H and ^{13}C NMR spectra of **5**.

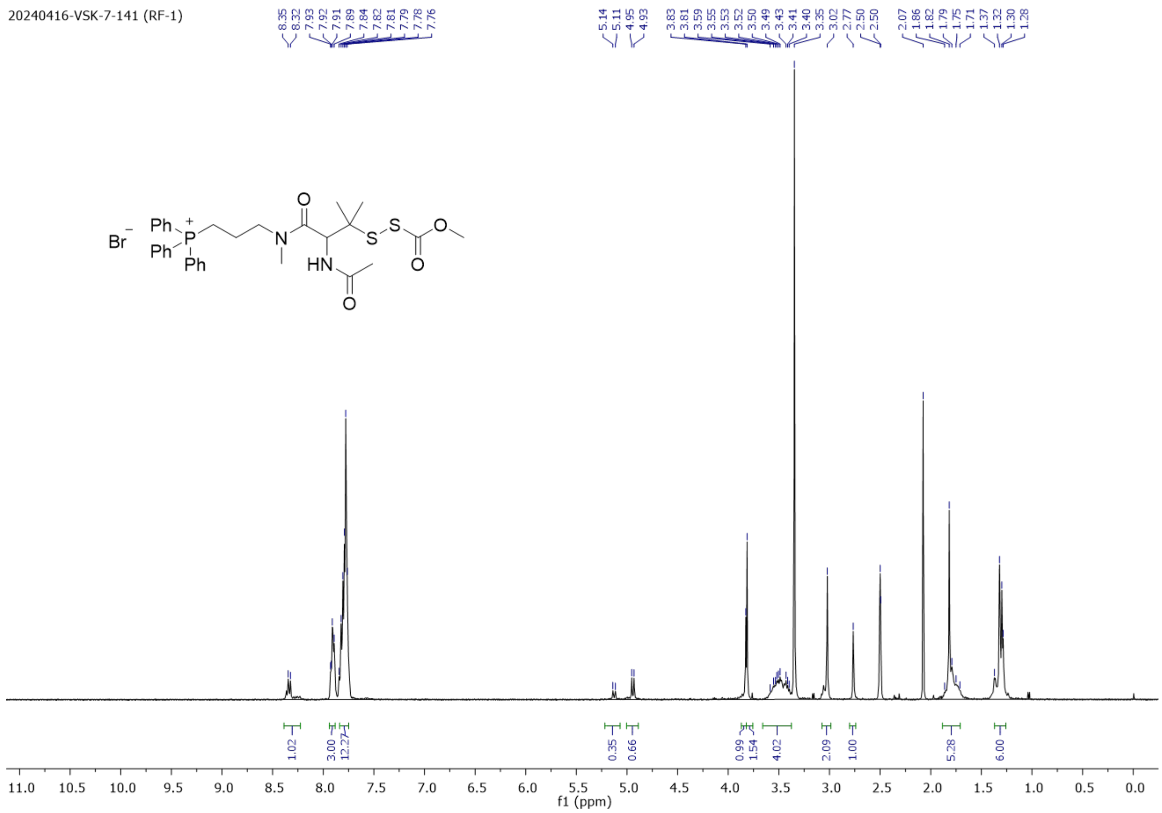

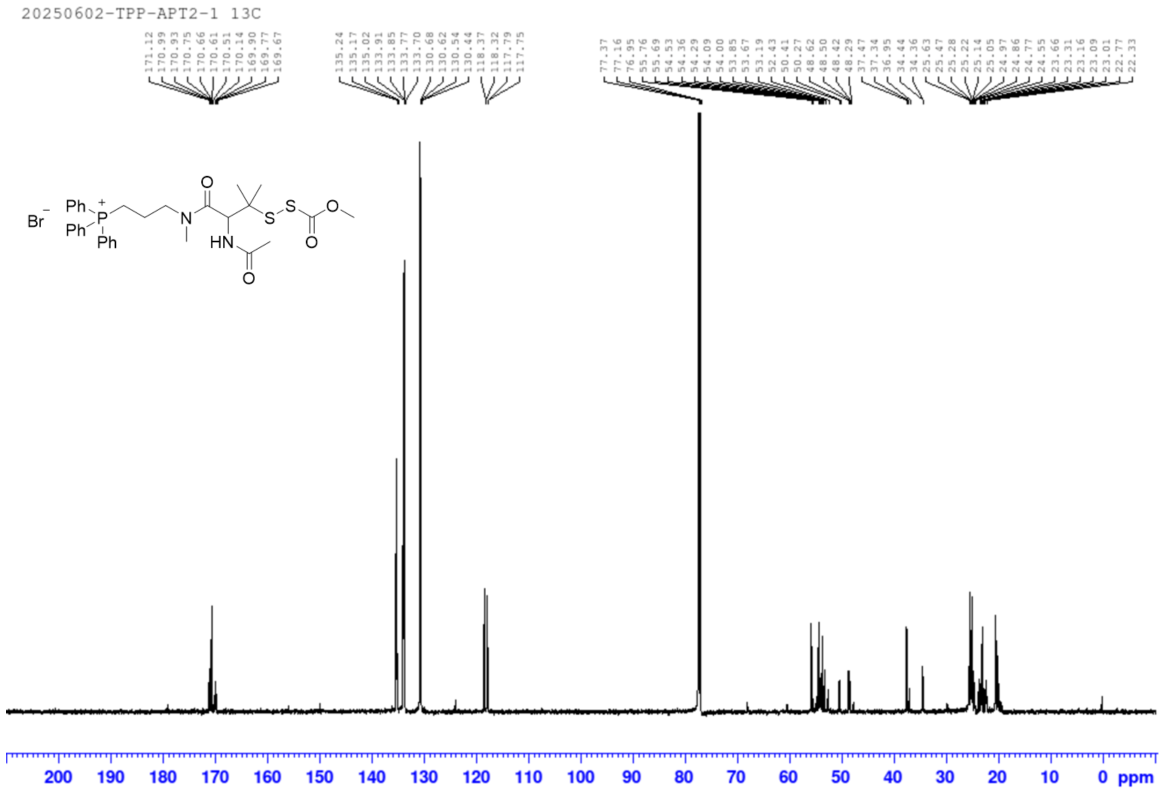

Figure S29. ^{1}H and ^{13}C NMR spectra of **AST-2-TPP**.

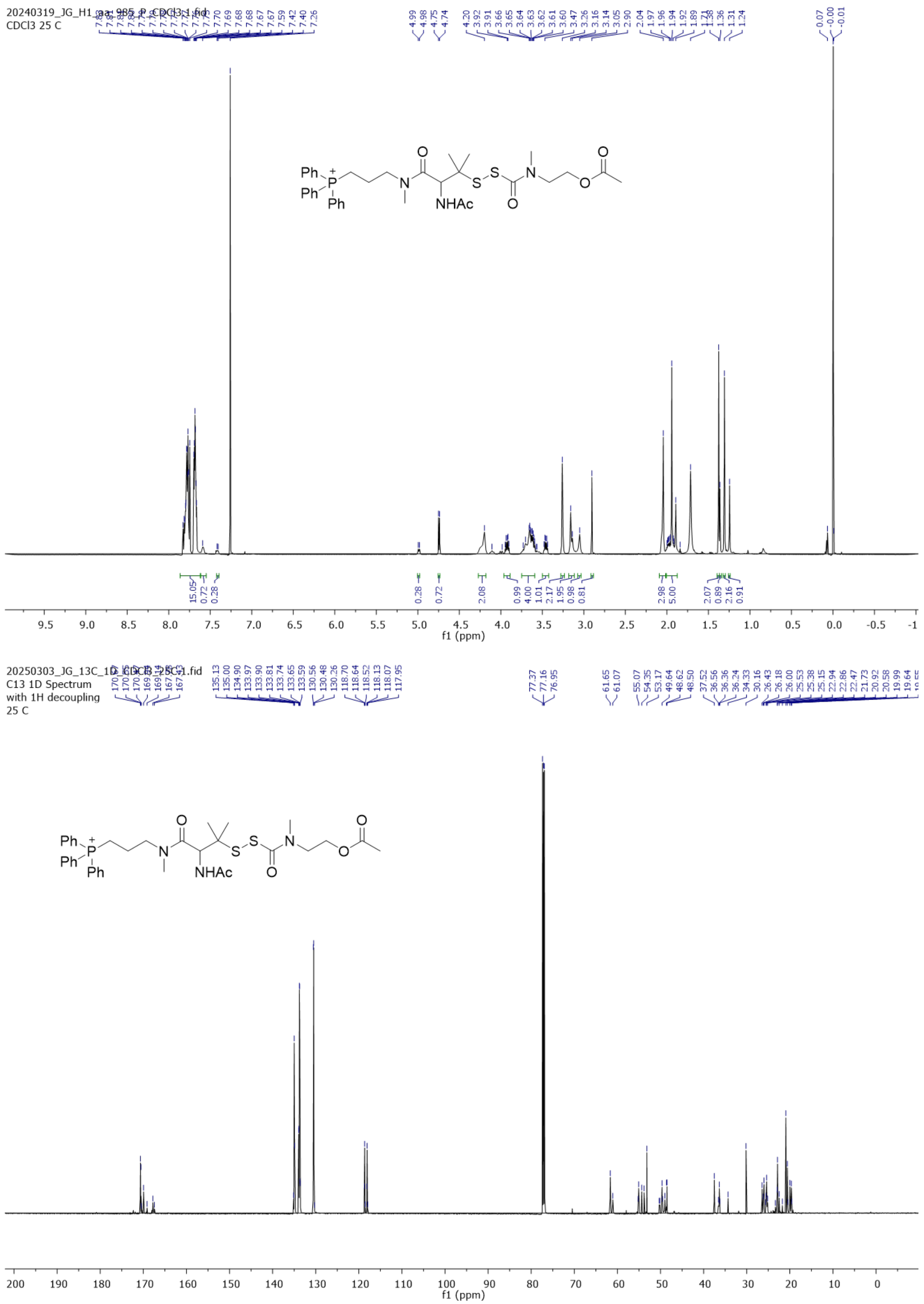

Figure S30. ^1H and ^{13}C NMR spectra of **APT-1-TPP**.

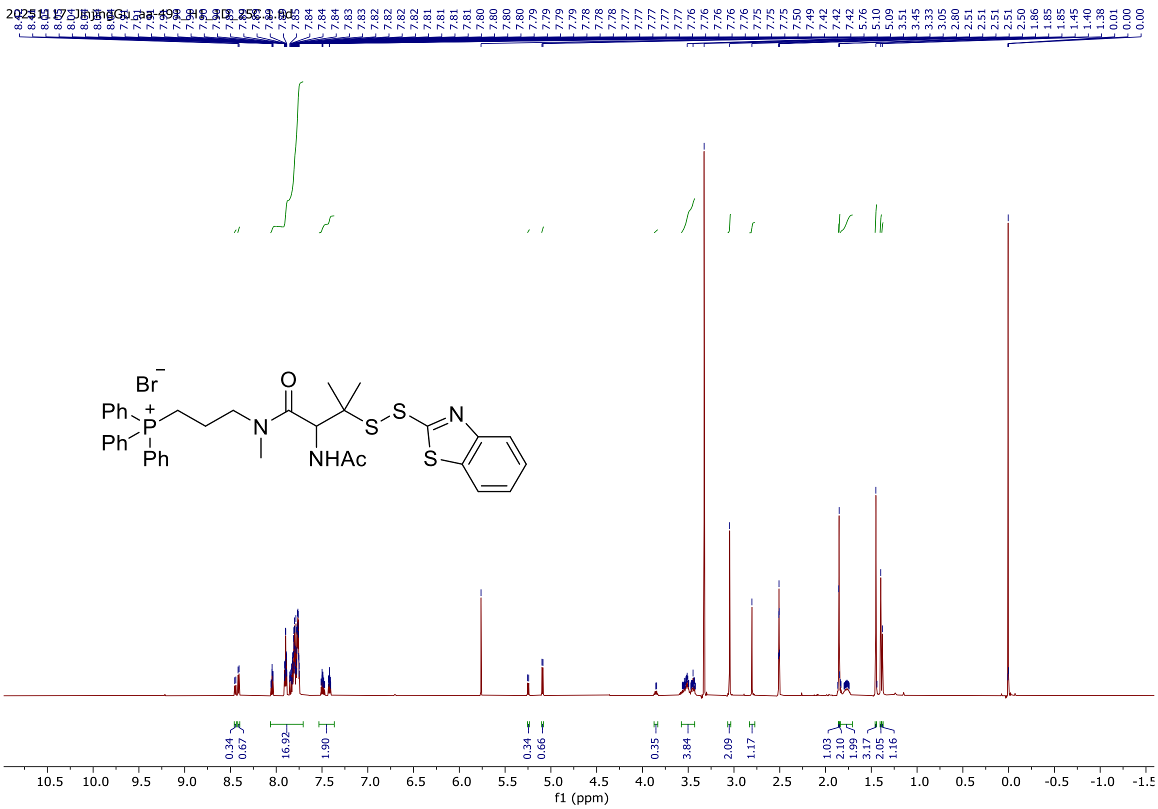

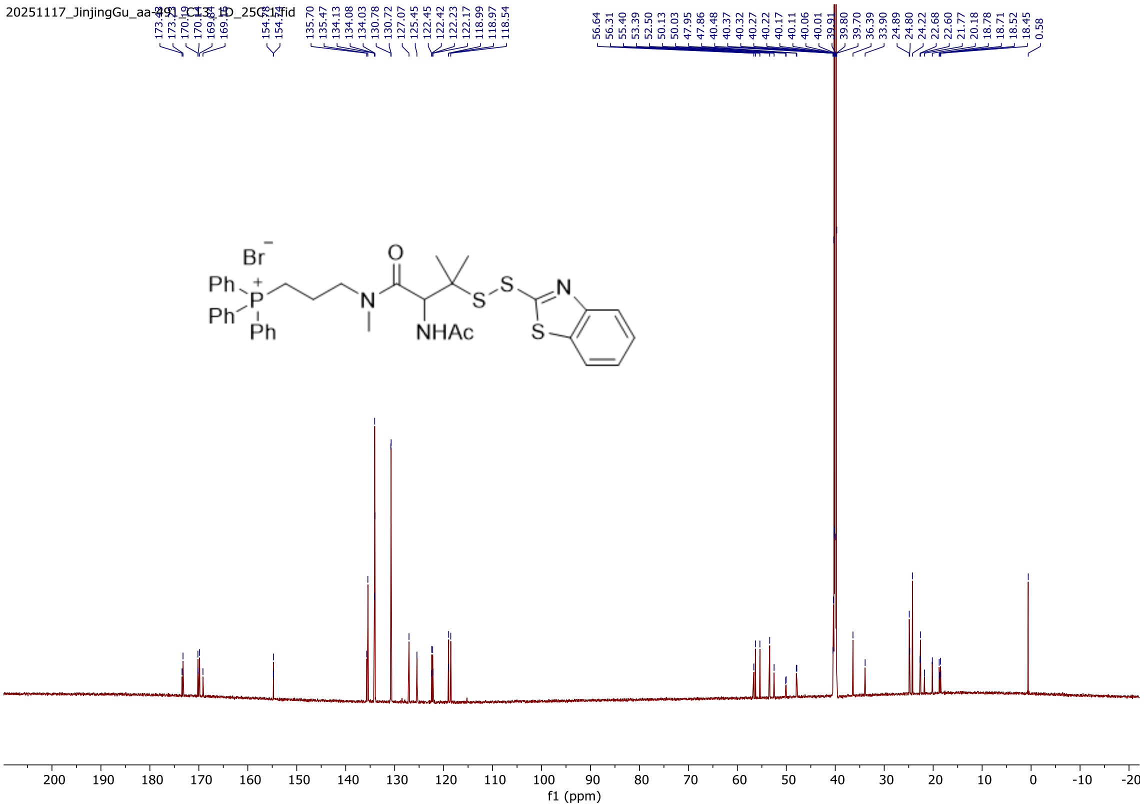

Figure S31. ^{1}H and ^{13}C NMR spectra of **compound S1**.

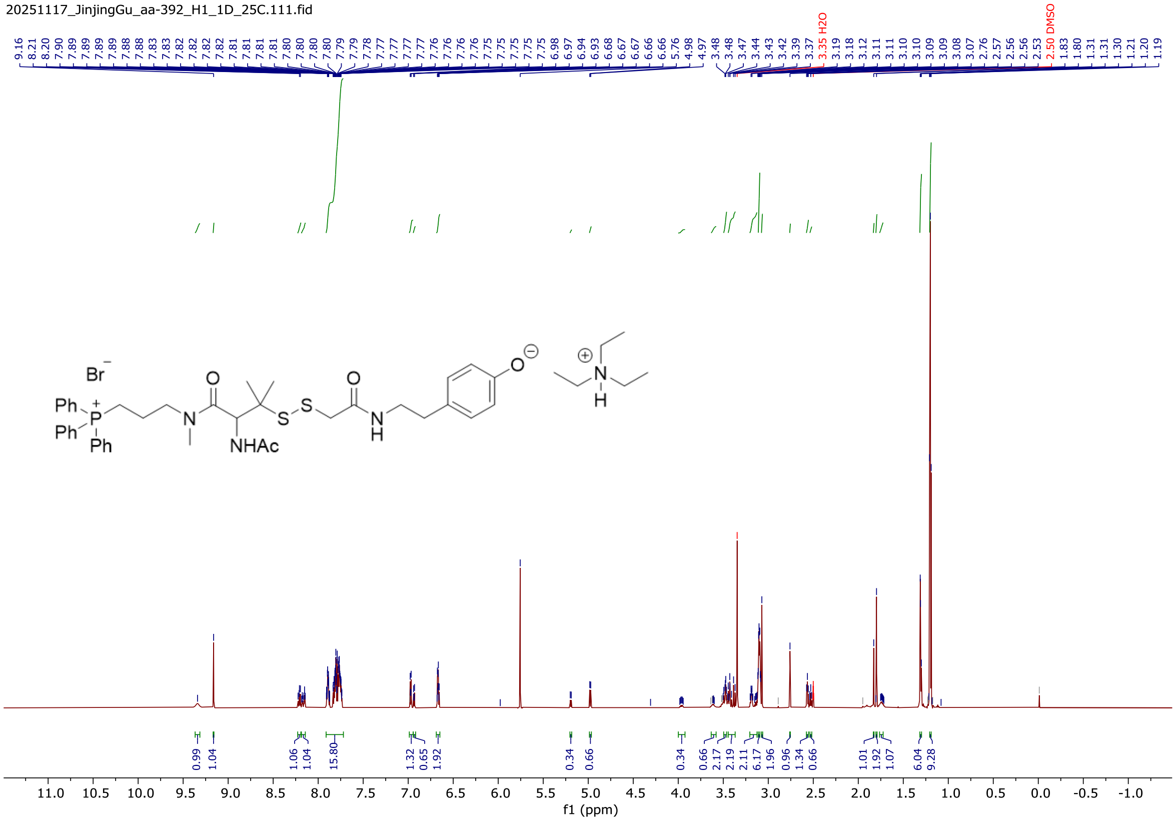

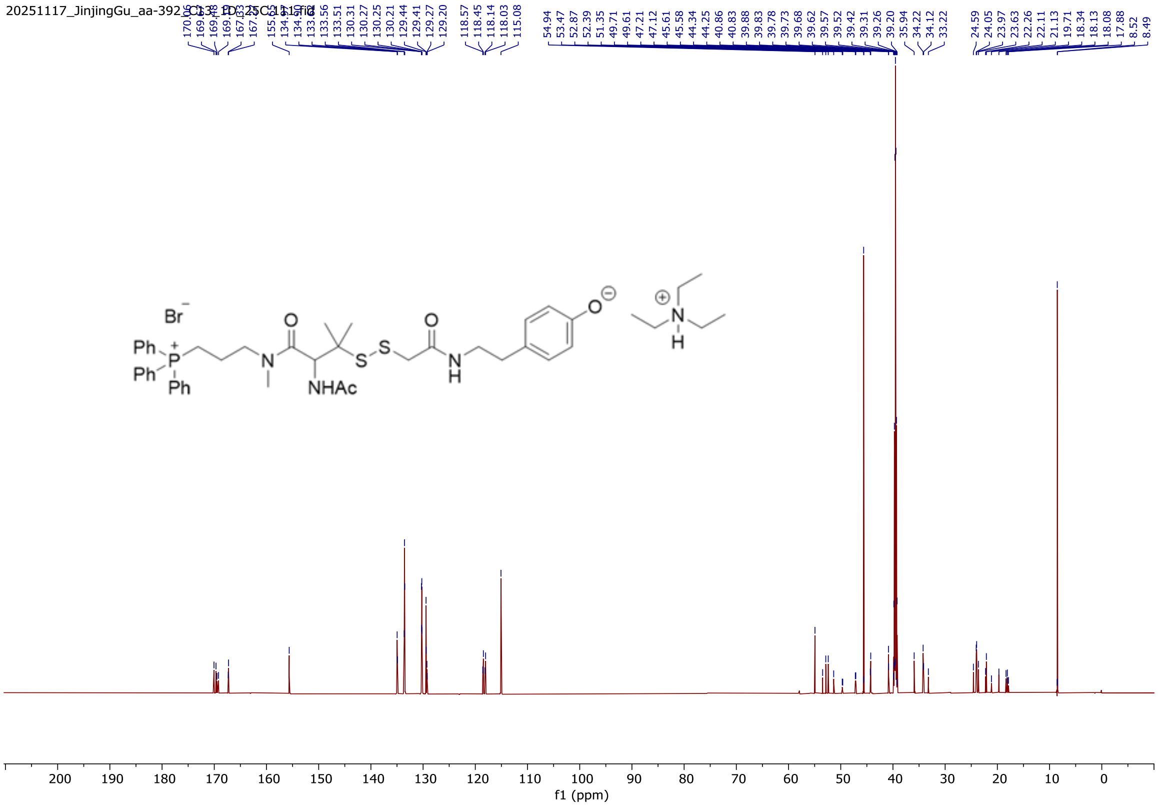

Figure S32. ^1H and ^{13}C NMR spectra of **TPP-RSS-HPE-AM**.
